# Charge transfer as a mechanism for chlorophyll fluorescence concentration quenching

**DOI:** 10.1073/pnas.2210811120

**Published:** 2023-01-23

**Authors:** Susannah Bourne-Worster, Oliver Feighan, Frederick R. Manby

**Affiliations:** ^a^Centre for Computational Chemistry, School of Chemistry, University of Bristol, Bristol BS8 1TS, United Kingdom; ^b^Entos, Inc., Los Angeles, CA 90027

**Keywords:** concentration quenching, chlorophyll, symmetric charge separation, environmental influences, Marcus theory

## Abstract

Highly concentrated solutions of chlorophyll display rapid fluorescence quenching. The same devastating energy loss is not seen in photosynthetic light-harvesting antenna complexes, despite the need for chromophores to be in close proximity to facilitate energy transfer. A promising, though unconfirmed mechanism for the observed quenching is energy transfer from an excited chlorophyll monomer to a closely associated chlorophyll pair that subsequently undergoes rapid nonradiative decay to the ground state via a short-lived intermediate charge-transfer state. In this work, we make use of newly emerging fast methods in quantum chemistry to assess the feasibility of this proposed mechanism. We calculate rate constants for the initial charge separation, based on Marcus free-energy surfaces extracted from molecular dynamics simulations of solvated chlorophyll pairs, demonstrating that this pathway will compete with fluorescence (i.e., drive quenching) at experimentally measured quenching concentrations. We show that the rate of charge separation is highly sensitive to interchlorophyll distance and the relative orientations of chromophores within a quenching pair. We discuss possible solvent effects on the rate of charge separation (and consequently the degree of quenching), using the light-harvesting complex II (LH2) protein from *rps. acidophila* as a specific example of how this process might be controlled in a protein environment. Crucially, we reveal that the LH2 antenna protein prevents quenching, even at the high chlorophyll concentrations required for efficient energy transfer, by restricting the range of orientations that neighboring chlorophyll pairs can adopt.

## Significance

The light-absorbing molecules (chlorophyll) in photosynthetic organisms must be held close together to achieve efficient energy transport but in solution, such close proximity leads to rapid energy loss (quenching). The hypothesized quenching mechanism is photoinduced charge separation followed by rapid charge recombination but this has never been proven. We confirm the feasibility of this mechanism using detailed calculations to show that charge separation outcompetes fluorescence (i.e., induces quenching) at chlorophyll separations compatible with the concentrations at which quenching is observed. Moreover, we reveal that the stiff photosynthetic protein environment inhibits quenching by preventing chlorophyll pairs from adopting a suitable shape for charge transfer. This insight into the protein function identifies a crucial design feature for efficient future artificial light-harvesting devices.

Chlorophyll molecules in photosynthetic light-harvesting complexes are surprisingly immune to the rapid fluorescence quenching displayed by concentrated solutions of chlorophyll in molecular solvents (1). The effect has consequently not been given much attention in studies of natural light harvesting (2). However, it highlights a fundamental role of the protein environment in tailoring the behavior of embedded chromophores for efficient energy transfer. Understanding first how this “concentration quenching” occurs and, second, how it is avoided in photosynthetic protein complexes should not be further overlooked in the context of developing new synthetic proteins (3) and biomimetic light-harvesting materials (4, 5), since protection against this unproductive quenching pathway must necessarily be a critical design consideration.

Measurements of fluorescence quenching in solutions of chlorophyll in both diethyl ether and acetone, reported in ref. (1), sparked several investigations into possible quenching mechanisms. Careful experiments by Porter et al. excluded the possibility of quenching due to trap states formed by impurities; intersystem crossing to the triplet state; and multiexcitation annihilation or collisions with quenching species (6). Unlike many other porphyrin-based pigments, chlorophyll does not readily aggregate in polar organic solvents (e.g., ethanol, ether) (7), as evidenced by the lack of concentration-dependent spectral changes (6, 8), so this was also ruled out as a potential cause of quenching.

The fluorescence decay kinetics of chlorophyll solutions suggest that quenching involves excitation transfer from an excited monomer to a (relatively) nonfluorescent dimer that acts as a trap (9). These nonfluorescent “dimers” are widely accepted to be statistical pairs: pairs of molecules that, in the random distribution in solution, happen to be (temporarily) within some critical distance of each other (8, 10). Beddard and Porter estimated this critical distance as 10Å by fitting experimental quenching data with Monte Carlo simulations of excitations performing random walks around randomly arranged 3D arrays of molecules (10). Their model used experimentally parameterized rates for fluorescence, intersystem crossing, and resonant energy transfer and assumed instantaneous quenching at statistical pairs.

The means by which excitations are trapped and quenched at statistical pairs is still unknown. The most promising hypothesis is that the excited chlorophyll pair undergoes a charge transfer ${\left(\;\text{Chl}-\;\text{Chl}\right)}^{*}\to {\;\text{Chl}}^{+}-{\;\text{Chl}}^{-}$ to form an ion pair that recombines back to the ground state (2, 7, 11). Electrostatic attraction between the intermediate ions may pull them closer together, helping to stabilize the pair and make it easier to reach the ground state. However, there is not yet any experimental evidence for the existence of an ion-pair intermediate (12).

An alternative possibility is that the chlorophyll molecules within a statistical pair form a temporary H-aggregate, where the lowest excited state has an oscillator strength close to zero and acts as a dark trap state (13, 14). Density functional theory calculations on chlorophyll pairs in vacuum support the idea that closely associated cofacial pairs do have a lower energy dark state (13) but the expected shift in absorption wavelength for the higher energy bright state is not observed experimentally (12). For this reason, we consider it the least likely of the two hypothesized mechanisms.

Nonradiative decay via an intermediate charge-transfer state is an established quenching mechanism for other small chromophores (15), bound porphyrin-based dyads (16), and chlorophyll in combination with carotenoids (17), where there is a redox potential between the molecules in the statistical pair. It is less clear whether the same mechanism would still operate between homogeneous chlorophyll pairs, although the question of symmetric charge transfer is of great interest in the context of understanding charge separation in the special pair of the photosynthetic reaction center. Theoretical studies of the special pair in vacuum indicate that there is a charge-transfer state energetically close to the first excited state (18, 19), although this could potentially change in the reaction center protein or in solution. Charge transfer has been observed in experimental models of the special pair (covalently linked cofacial porphyrin units) (20, 21) and is known to drive quenching in side-by-side bacteriochlorin or porphyrin dyads (16, 22, 23). However, it is not known to what degree the covalent linking in these models might influence their charge-transfer properties. Charge transfer in these linked systems is more pronounced in solvents with a high dielectric constant, implying that chlorophyll concentration quenching, if it proceeds via a charge-transfer mechanism, should have a solvent dependence. This is loosely in agreement with early experimental results (1), although more data would be needed to confirm the trend.

Our aim in this paper is to provide concrete evidence for the feasibility of a quenching mechanism involving charge transfer by calculating the free energy surfaces (FESs) for the photoexcited and intermediate charge-separated state in a solvent environment. We calculate the rate of charge separation at chlorophyll separations around the predicted “critical separation” and compare these to the fluorescence rate to determine the likelihood of observing significant quenching. Finally, we investigate how these surfaces change in a protein environment, revealing how photosynthetic antenna proteins may inhibit quenching to allow efficient energy transport.

## Results

**Fig. 1.**
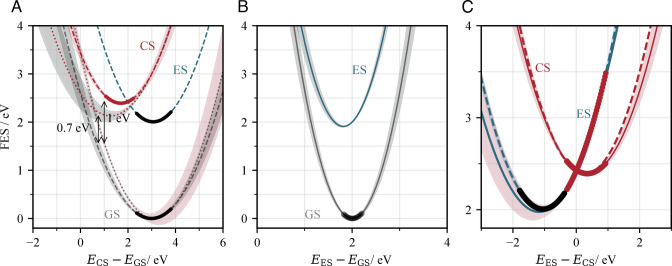
Relationships between (*A*) The charge-separated ${\;\text{Chl}}^{+}-{\;\text{Chl}}^{-}$ state (red) and ground Chl – Chl state (gray), (*B*) The photoexcited ${\left(\;\text{Chl}-\;\text{Chl}\right)}^{*}$ state (blue) and ground state (gray), and (*C*) The charge-separated state (red) and photoexcited state (blue) for a 10Å-separated pair of chlorophyll molecules in diethyl ether. Each pair of states are plotted as a function of the energy gap between them. The dashed charge-separated and ground state surfaces are constructed using Eq. **3**, based on the distribution of energy gaps at the sampled points (indicated with a thicker line). The solid blue photoexcited state surface in *B* and *C*. and the dotted red and gray surfaces in *A* are constructed by adding the energy gap (along the x-axis) to the energy of the other state in the pair. In *A*, the photoexcited state is also plotted (blue dashed line) as a function of the energy gap between the ground and charge-separated states. This surface is created using the fitted linear relationship between the ES-CS and GS-CS energy gaps (*SI Appendix*). The dashed red CS surface and dashed blue ES surface in *A* are replotted in *C* (dashed red and blue lines respectively) as a function of $E_{\;\text{ES}}-E_{\;\text{CS}}$. Shaded regions around each surface indicate the statistical uncertainty arising from the fitting procedure used to create them (*SI Appendix* for details of how these uncertainty regions are determined). The color of the shading indicates which fitting procedure led to the uncertainty: red shading is associated with fitting the distribution of energy gaps along the charge-separated MD trajectory, grey with fitting the distribution of energy gaps along the ground state trajectory and blue with fitting the relationship between ES-CS and GS-CS energy gaps.

Quenching via a charge-separated state involves three states of the statistical pair of chromophores: the initial photoexcited state ${\left(\;\text{Chl}-\;\text{Chl}\right)}^{*}$, the intermediate charge-separated state ${\;\text{Chl}}^{+}-{\;\text{Chl}}^{-}$, and the ground-state Chl – Chl. Fig. 1 shows the free-energy surfaces (FESs) for each pair of these states as a function of the energy gap between them, which takes the place of a reaction coordinate in collectively describing fluctuations in the molecular and solvent geometry. The surfaces are reconstructed from the Boltzmann distribution of energy gaps (reflecting the distribution of thermally accessible geometries) sampled over an equilibrated molecular dynamics trajectory, as detailed in *Materials and Methods*. The energy gap distributions that we obtain (*SI Appendix*, Figs. S2 and S3) are well fitted by a normal distribution, indicating that the underlying free-energy surfaces are quadratic around their minimum. We initially make the assumption that this quadratic form can be extrapolated out to unsampled regions of the configuration space. This is consistent with the quadratic form of free-energy surfaces that have previously been calculated for bacteriochlorophyll pairs in the photosynthetic reaction center of *Rb. Sphaeroides* (24). However, Fig. 1*A* highlights the potential problem with extrapolating the quadratic surfaces too far beyond the sampled region. Away from the sampled region of a FES (indicated by a thicker line in Fig. 1), the predicted energy difference relative to another state can be quite different from the values sampled by a second molecular dynamics simulation. For example, 1.5eV above the ground state (GS) minimum in Fig. 1*A*, the energy gap predicted by extrapolating the fitted GS surface to smaller energy gaps (the difference between the dashed gray line and dotted red line) is 0.7eV, whereas the energy gap obtained by directly fitting the sampled Boltzmann distribution for the charge-separated (CS) state (the difference between the dashed red line and dotted gray line) is 1eV. This disparity could indicate that the GS surface deviates from a purely quadratic form further from its minimum. It could also be a result of sampling uncertainty associated with fitting the Boltzman distribution of energy gaps, which introduces uncertainty into the shape of the corresponding FES (indicated by the shaded regions in Fig. 1). In either case, it indicates that we should exercise caution when extrapolating the fitted surfaces beyond the sampled region.

Within the Marcus theory framework that we will adopt, the rate constant for charge separation depends heavily on the position of the minimum of the photoexcited (ES) state. Since, due to the computational cost, we do not perform an excited state molecular dynamics simulation, the ES minimum cannot simply be determined as the peak of a sampled Boltzmann distribution, as can be done for the GS and CS states. Moreover, Fig. 1*C* indicates that the ES minimum is significantly displaced relative to the CS minimum, with the consequence that the important $E_{\;\text{ES}}-E_{\;\text{CS}}$ gap at the ES minimum is not sampled by molecular dynamics simulations of solvated chlorophyll ions (i.e., the charge-separated state). Previous studies of ground-state charge transfer have used a form of umbrella sampling to extend the sampled region of the charge-separated state, by running multiple MD simulations with different amounts of partially transferred charge (25). The same solution is not so easily implemented in this case (although we note that a more naive biasing potential may prove effective) since it would involve creating a forcefield for a chlorophyll pair that is partially in an excited state. However, since the ES surface is only slightly displaced relative to the GS surface (Fig. 1*B*), molecular/solvent configurations around the ES minimum are sampled by MD simulations of the neutral, ground state chlorophyll pair. Around the GS minimum, $E_{\;\text{ES}}-E_{\;\text{CS}}$ correlates well with $E_{\;\text{CS}}-E_{\;\text{GS}}$ (*SI Appendix*, Fig. S6). By fitting the linear relationship between these two energy gaps, the ES surface can be defined relative to the CS surface as a function of $E_{\;\text{CS}}-E_{\;\text{GS}}$ (Fig. 1). Fig. 1*C* shows the same ES surface replotted as a function of $E_{\;\text{ES}}-E_{\;\text{CS}}$ (dashed blue line). We find that the shape of the ES surface (particularly the position of the minimum) defined in this way agrees fairly well with the ES surface predicted by extrapolating the CS surface (solid blue line), offering reassurance that the extrapolated quadratic form continues to be a good description of the CS state in this part of the configuration space. However, since the extrapolation introduces a greater amount of uncertainty, we use the ES surface derived from the ground-state molecular dynamics trajectory (i.e., the dashed blue line) to evaluate Marcus theory parameters in the following analysis.

### Charge Separation in a 10Å Separated Dimer.

**Fig. 2.**
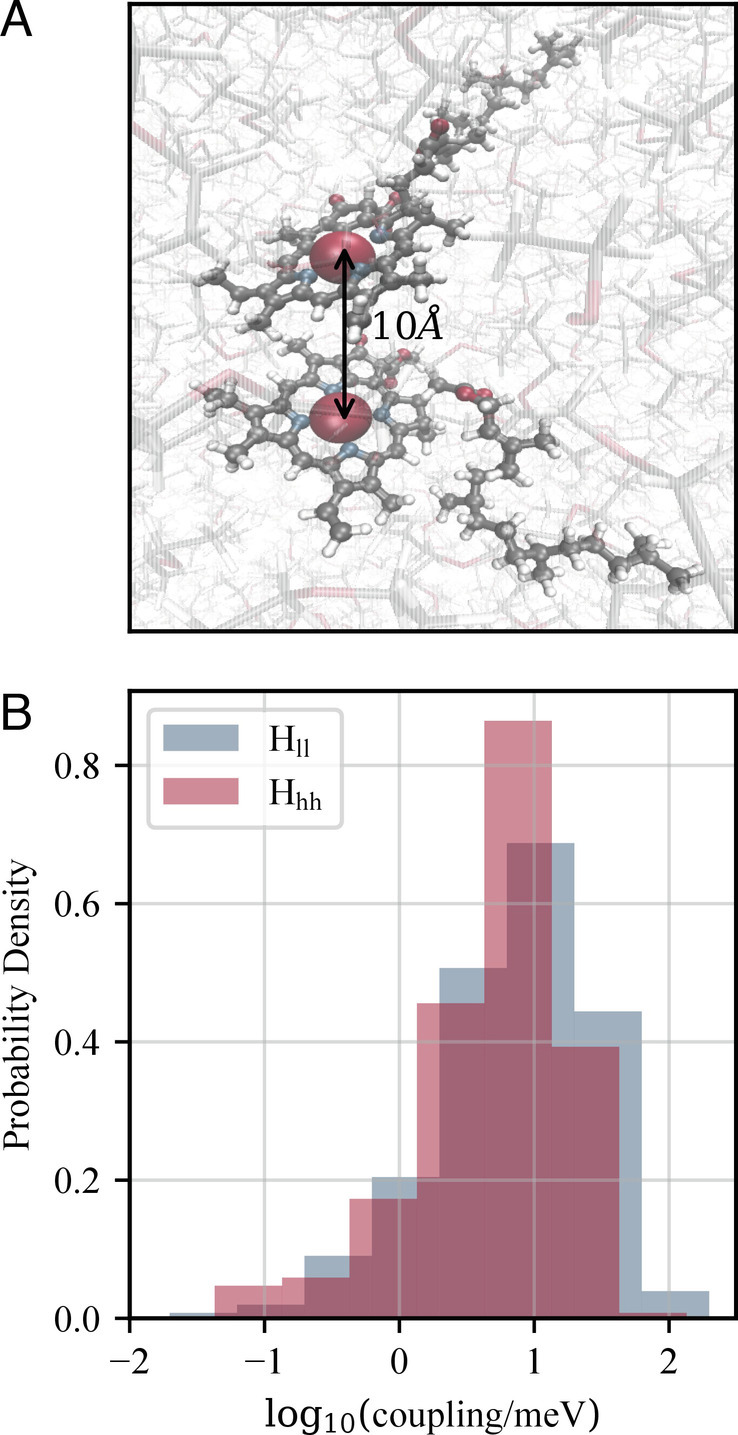
10Å separated chlorophyll pair. (*A*) Molecular dynamics simulations were performed for pairs of chlorophyll molecules solvated in diethyl ether. The Mg–Mg separation, indicated in the figure, was fixed as a single constraint in the forcefield. (*B*) Distribution of coupling values between the excited and charge-separated states, calculated using FODFT at the PBE/3-21++G level on chlorophyll geometries from 500 different frames of the neutral ground-state trajectory, spaced 1ps apart. Two different coupling values were calculated for each geometry, corresponding to coupling between the ground-state HOMO orbitals $H_{\;\text{hh}}$ or ground-state LUMO orbitals $H_{\;\text{ll}}$ on each chlorophyll ion fragment.

**Fig. 3.**
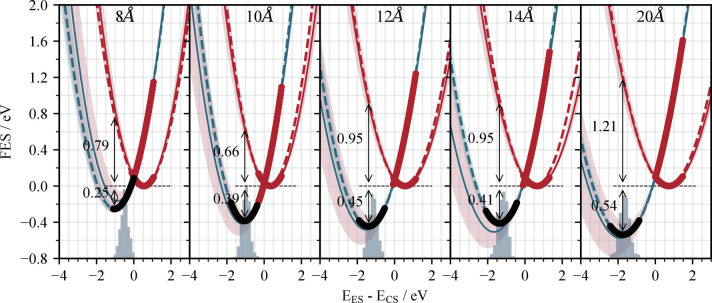
Distance dependence of the free-energy surfaces for the photoexcited (ES) ${\left(\;\text{Chl}-\;\text{Chl}\right)}^{*}$ state (red line) and charge-separated (CS) ${\;\text{Chl}}^{+}-{\;\text{Chl}}^{-}$ state (blue line) of chlorophyll pairs in a solution of diethyl ether. The Mg–Mg separation is indicated at the top of each panel. As in Fig. 1*C*, the solid red CS surfaces are derived from the distribution of energy gaps sampled along charge-separated MD trajectories using Eq. **3** and the corresponding solid blue line by simply adding the energy gaps (along the *x*-axis) to the solid CS surface. The dashed red line is derived from sampling $E_{\;\text{CS}}-E_{\;\text{GS}}$ along the charge-separated trajectory and the dashed blue line from sampling $E_{\;\text{CS}}-E_{\;\text{GS}}$ and $E_{\;\text{ES}}-E_{\;\text{CS}}$ along the ground-state trajectory, in the same way as shown in Fig. 1*A*. Sampled regions of the surfaces are indicated by a thicker line, with red showing regions that were sampled in the charge-separated trajectory and black showing regions that were sampled in the ground-state trajectory. The regions of uncertainty (shaded regions) are derived in the same way as for Fig. 1. The values of the free energy change and reorganization energy are derived from the dashed surfaces, since these have a significantly lower uncertainty (due to direct sampling of the ES minimum). The ground-state thermal distribution is illustrated by the blue histogram.

Beddard and Porter’s 1976 model (10) suggested that quenching occurs between pairs of chlorophyll molecules separated by 10 Å or less. Taking 10 Å to be the distance between the central magnesium atoms (Fig. 2*A*), we start by examining the energetics of charge separation at this postulated critical separation for a chlorophyll A pair solvated in ether (matching the experimental conditions in ref. (1)). Fig. 1*C* (reproduced in the second panel of Fig. 3) illustrates the ES and CS free-energy surfaces at this separation. Sampling uncertainty in the shape of these surfaces carries through into the Marcus theory parameters that we derive from them. In the following, we report the value of each quantity obtained from our original dataset, followed by a lower and upper bound on the value (*Lower, Upper*). These bounds reflect the full range of values produced by the 95% confidence interval on the statistical parameters used to construct the free-energy surfaces. A more detailed description of the procedure used to generate these uncertainty intervals is given in *SI Appendix*.

At this separation, the charge-separated state lies slightly higher in energy than the first excited state, with a free energy difference between the minima of the two surfaces of 0.39 [0.38, 0.39]eV. This is broadly in agreement with optical measurements of chlorophyll, ES energy = 1.9eV (26); CS energy = ionization energy + electron affinity = 2.6eV (27), although under different experimental conditions to those modeled here. The reorganization energy associated with the charge separation (the energy required to rearrange the chlorophyll molecules and their surrounding solvent from the optimal excited state geometry to the optimal geometry for the charge-separated state) is 0.66 [0.63, 0.71]eV.

The coupling $H_{\;\text{AB}}=\;\text{ES}|\hat{H}|\;\text{CS}$ between the donor (ES) and acceptor (CS) states for the charge separation depends on the relative orientation of the two chlorophyll molecules and consequently takes a wide range of values in solution, as shown in Fig. 2*B*. The values of $H_{\;\text{AB}}$ were obtained using Fragment Orbital Density Functional Theory (*Materials* *and* *Methods*), which approximates the coupling between the overall donor and acceptor states as the coupling between the donor and acceptor orbitals of the isolated molecules. To avoid making assumptions about the direction of the charge transfer, we consider both the coupling $H_{\;\text{hh}}$ between the HOMOs (highest occupied molecular orbital in the neutral groundstate) on each molecule, which corresponds to Chl${}^{*}$ – Chl→ Chl${}^{-}$ – Chl${}^{+}$ and the coupling $H_{\;\text{ll}}$ between the LUMOs (lowest occupied molecular orbitals), which corresponds to Chl${}^{*}$ – Chl→ Chl${}^{+}$ – Chl${}^{-}$. The coupling strength at a given geometry of the chlorophyll pair does not correlate well with the energy gap between the ES and CS states (*SI Appendix*, Fig. S7), making it difficult to pinpoint the appropriate value of the coupling at the crossing point. Since our aim is to explore the feasibility of charge separation as a quenching mechanism, we use the most optimistic (largest) value $H_{\;\text{ll}}=0.115$eV in the following analysis.

Using the Marcus theory equation for the rate of electron transfer,

[1] $$\begin{array}{c} k_{\;\text{et}}=\frac{2\pi }{\hbar }{\left|H_{\;\text{AB}}\right|}^{2}\frac{1}{\sqrt{4\pi \lambda k_{\;\text{B}}T}}\exp(\frac{-{\left(\lambda +\Delta A\right)}^{2}}{4\lambda k_{\;\text{B}}T}), \end{array}$$

we obtain a rate constant $k_{\;\text{CS}}$ for the charge separation of 0.03 [0.02, 0.04]ns^−1^. This suggests that charge separation should be noticeable on the timescale of chlorophyll fluorescence, which occurs with a rate constant $k_{\;\text{f}}$ on the order of 0.2ns^−1^ the fluorescence lifetime for chlorophyll *a* in ether is 5.1ns (28), although Beddard and Porter assume a slower rate constant of $k_{\;\text{f}}=0.01\;{\;\text{ns}}^{-1}$ in their original model (10). However, it may not be sufficiently fast to outcompete fluorescence and lead to significant quenching. Eq. **1** assumes that the photoexcited chromophores (and surrounding solvent) have fully relaxed to their new equilibrium geometry before charge separation commences. It is also worth considering that the initial rate constant for the charge separation $k_{\;\text{CS}}^{\;\text{init}}$ may be larger than the eventual equilibrium value calculated above. We can estimate $k_{\;\text{CS}}^{\;\text{init}}$ using Eq. **1** with a reorganization energy and free-energy difference corresponding to the geometry distribution immediately after photoexcitation (29) (i.e., the ground-state thermal distribution), shown as blue histograms on Fig. 3. With these values (λ= 0.63eV and ΔA= 0.39eV), $k_{\;\text{CS}}^{\;\text{init}}=$ 0.04 ${\;\text{ns}}^{-1}$. However, since vibrational relaxation likely occurs faster than either fluorescence or charge separation (rate constant estimated at 8.5ps^−1^ (30)), we consider that the equilibrated rate constant provides a better description of the charge separation process. In the remainder of our analysis, we only consider the Marcus theory parameters at the relaxed excited state configuration.

### Effect of Chl – Chl Separation.

Increasing the distance between chlorophyll magnesium centers decreases the rate of photo-induced charge separation. Fig. 3 shows the change in the ES and CS surfaces over a range of separation distances, from 8 Å (well within the postulated critical quenching separation) to 20 Å (essentially noninteracting). Both the reorganization energy and the free energy difference ΔA increase systematically toward a limiting value with increasing Mg–Mg separation, leading to a visible increase in the height of the crossing point as the two chlorophyll molecules get further apart. The rate of charge separation, which depends exponentially on ${\left(\Delta ,A,+,\lambda \right)}^{2}$, consequently decreases by 2 orders of magnitude as the Mg–Mg distance is increased from 8 Å to 10 Å, even assuming a constant value for the coupling between the two states. A further order of magnitude decrease in the rate is seen on reaching a 12 Å separation.

The coupling $H_{\;\text{AB}}$ is proportional to the overlap of the donor and acceptor orbitals, which, in this case, are the same orbital centered on different residues. The two-center overlap integral between Slater orbitals has a complicated dependence on the center-to-center separation R but, at long distances, decays approximately as exp(−nR) (31), with $n={\left(-2\epsilon \right)}^{\frac{1}{2}}$ (32), where ϵ is the smallest (absolute) occupied orbital energy. Chlorophyll has an ionization energy (−ϵ according to Koopman’s theory) of 6.5eV, giving n=1.3Å^−1^. This is in excellent agreement with systematic studies of the distance dependence of $H_{\;\text{AB}}$ for porphyrin rings (33). Thus, we expect the coupling to decrease by a factor of 13 with each additional 2 Å separation of the chlorophyll residues. This corresponds to another two orders of magnitude decrease in $k_{\;\text{CS}}$. Combined with the distance dependence of the reorganization energy and the free-energy difference, we conclude that the rate of charge separation decays rapidly with increasing chlorophyll separation.

### Charge Separation in LH2.

Previous studies have hypothesized that photosynthetic antenna complexes avoided unwanted concentration quenching by holding chromophores sufficiently far apart (8, 10). The Fenna–Matthews–Olson (FMO) complex was considered optimally designed, since the average chromophore separation (12 Å) is only fractionally greater than the postulated critical quenching separation (10 Å), maximizing exciton transport whilst inhibiting quenching. This argument is not so easily transferred to light-harvesting complex 2 (LH2) in purple bacteria (*Rhodopseudomonas acidophila*), where the average bacteriochlorophyll separation in the B850 ring is only 9 Å.

**Fig. 4.**
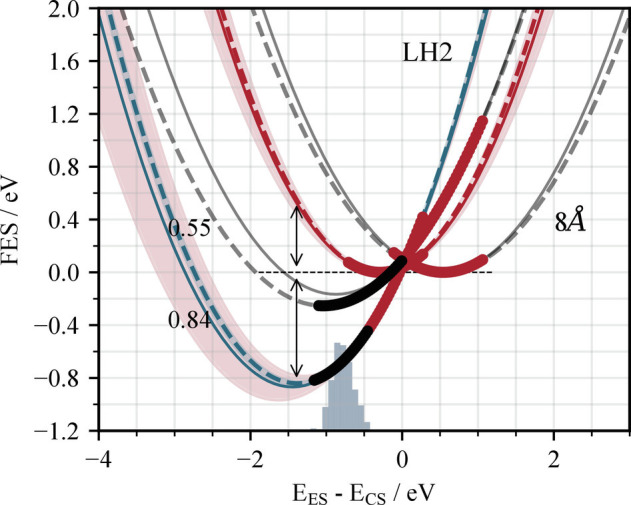
Free-energy surfaces for the photoexcited ${\left(\;\text{BChl}-\;\text{BChl}\right)}^{*}$ state (dashed red line) and charge-separated ${\;\text{BChl}}^{+}-{\;\text{BChl}}^{-}$ state (dashed blue line) of a pair of neighboring chlorophyll molecules in the B850 ring of light-harvesting complex 2 (LH2). The equivalent surfaces for a chlorophyll pair in diethyl ether with a 8Å separation (Fig. 3) are shown in gray for comparison. The surfaces and their regions of uncertainty (shaded regions) are derived in the same way as for Fig. 2*B*. The values of the free-energy change and reorganization energy are given for LH2, as is the ground-state thermal distribution, illustrated by the blue histogram.

Fig. 4 shows the free-energy surfaces for the excited and charge-separated states of a neighboring pair of bacteriochlorophyll chromophores in the B850 ring. It is instantly noticeable that these surfaces are steeper than the corresponding surfaces for chlorophyll dissolved in ether, indicating that the protein environment significantly restricts the number of thermally accessible geometries that chlorophyll can adopt. The charge-separated state is destabilized compared to the photoexcited state (ΔA= 0.84 [0.83, 0.86]eV), and its minimum is shifted to the left (λ= 0.55 [0.53, 0.58]eV). The crossing point from the ES to the CS state is higher in energy than even the 20Å separated pair in ether. Consequently, the rate of charge separation is negligible. Furthermore, the ES state crosses the CS state very close and slightly to the right hand side of its minimum (placing the reverse process in the normal rather than inverted Marcus region). This suggests that, even with sufficient energy to reach the crossing point, the system would quickly return to the excited state.

### Returning to the Ground State.

The lack of spectroscopic evidence for a charge-separated intermediate suggests that this state, if it forms at all, is extremely short-lived. It has previously been suggested that this implies the ground state crosses the charge-separated state close to the latter’s minimum. Fig. 1*A* clearly shows that this is not the case. Using Eq. **1** to calculate the charge recombination rate constant similarly implies that recombination from the charge-separated state would be extremely slow. However, chlorophyll pairs do not arrive in the charge-separated state with the thermal distribution of geometries assumed by Marcus theory but rather with the geometries associated with crossing over from the excited state. Therefore, we do not consider Marcus theory an appropriate approach to describing rapid recombination from the charge-separated state. Charge recombination could be extremely fast if the molecular and solvent configuration for the initial charge separation places the system close to the crossing point between the charge-separated and ground states. The large, negative free energy change on decaying to the ground state then ensures that the process is effectively irreversible. Fig. 1 *A* and *C* suggests that the ES→ CS crossing point at least places the system energetically closer to the CS→ GS crossing point than if it started from the CS minimum. Furthermore, comparing Fig. 1 *A* and *B* shows us that the charge-separated state, although higher in energy than the excited state, crosses the ground state at a lower energy point (for the best comparison, we consider the dashed red and dotted gray lines in Fig. 1*A*). This indicates that returning to the ground state via the charge-separated state is more favorable than direct decay from the photoexcited state. If small fluctuations in the relative angle of the porphyrin head groups reduce the Mg–Mg separation (not permitted within our simulations but likely favored by the electrostatic attraction between the oppositely charged ions), returning to the ground state becomes even easier. However, a proper evaluation of the recombination process requires a more detailed dynamical analysis that is beyond the scope of the present study.

## Discussion

### Charge Separation.

We have shown that charge separation between chlorophylls dimers competes with fluorescence at separations of approximately 10Å and less and, furthermore, that it is easier to return to the ground state from a charge-separated state than from the original photoexcited state. This supports the hypothesis that fluorescence quenching in concentrated chlorophyll solutions is driven by nonradiative decay via an intermediate charge-transfer state, although further work is needed to confirm that the second step of the mechanism (charge recombination) is also sufficiently fast.

The rate constant for charge separation $k_{\;\text{CS}}$ decreases smoothly with increasing Mg–Mg distance, contrary to previous theoretical models that have assumed a critical “cutoff” distance below which quenching occurs and is instantaneous. However, because $k_{\;\text{CS}}$ decreases extremely quickly, using a cutoff distance is not a bad assumption: the dominating process for a nearby pair of chlorophyll molecules will switch from fluorescence to charge separation over the space of just a few angstrom. Consequently, there is a good agreement between the cutoff distances of earlier studies (10) and the distances at which charge separation becomes a competitive process in the current work.

In reality, we expect the agreement to be even closer as the rate constants that we predict are likely an underestimate. First, the forcefield and point-charge embedding used to describe the ether solvent are not polarizable. Therefore, they are likely to overestimate the polarization of the atomic point charges in the forcefields constructed for the ion pairs and consequently the destabilization of the CS state compared to the ES state. This is less likely to be an issue for the LH2 FESs in Fig. 3, since this system was simulated using a recently developed, high-quality, polarizable forcefield (34) (*Materials and Methods*). Second, FODFT is known to increasingly underestimate coupling values with increasing distance (due to basis set truncation errors), although in this case, this will be at least partially offset by the fact that we have chosen the most optimistic coupling values for calculating the rate constant.

Propagation of statistical uncertainty in the shape of the free-energy surfaces introduces some uncertainty into the associated Marcus theory parameters and charge-transfer rate constants, although direct sampling of energy gaps around the CS and GS minimum help to keep this relatively small. The low-level semiempirical methods that we employ add yet another source of uncertainty. However, both GFN1-xTB and the bespoke method used for local excitations have an excellent ability to describe the small relative energy changes associated with fluctuations in geometry, meaning that they should correctly capture the shape of the energy gap distributions that we use to derive free-energy surfaces. Consequently, we consider uncertainty in the underlying electronic structure methods to be a minor effect. The ability of these methods to accurately capture the absolute values of the energy gaps between different electronic states is potentially a bigger concern, although the reasonable agreement with experimental data approximating this energy gap (26, 27) suggests that the error is relatively small. The sensitivity of calculated charge separation rate constants to even small changes in these energy gaps suggests that the quantitative results of this study should be treated with caution. Nonetheless, the fact that we predict charge separation will outcompete fluorescence by several orders of magnitude for the shortest separation investigated (8Å) inspires confidence in the qualitative conclusion that this is likely to be a feasible mechanism for concentration quenching. Similarly, the drastically different shape of the free-energy surfaces for BChl pairs in LH2 allows us to make qualitative observations about the role of the protein complex in tailoring the behavior of chromophore pairs. The low computational cost of semiempirical methods was critical in enabling the broad investigation of different chlorophyll and solvent geometries in this study. It is our hope that the qualitative overview that we present will provide a springboard to target more costly, higher-level investigations into the details of this mechanism.

This includes investigations into the nature of the intermediate state. It is possible that the intermediate state is actually an excited state with some degree of charge-transfer character, rather than a pure charge-separated state (16, 22). This possibility could be explored further using a charge-transfer exciton Hamiltonian (35, 36). However, we note that the factors that affect relative state energies will also affect the degree of mixing between the excited and charge-separated states. Therefore, although a more detailed analysis of the character of the intermediate state might give a quantitatively different prediction of how quickly it forms, we would, again, not expect it to change the qualitative trends discussed in this study.

### Ensemble Rates and Aggregation.

For efficient overall quenching, there need to be a sufficient number of chlorophyll pairs forming in solution at separations where quenching competes with fluorescence. A back-of-the-envelope calculation reassures us that at a concentration of 0.014mol dm^−3^ (50% quenching (1)) 4% of chlorophyll molecules will be in pairs with a center-to-center separation of 10Å or less, with half of those pairs having a separation of 8Å or less. This assumes a uniform distribution of chlorophyll molecules and an excluded volume for chlorophyll of $4\pi r_{g}^{3}/3$, where $r_{g}=0.96$nm is the radius of gyration (*SI Appendix*). At 0.1mol dm^−3^ (≈100% quenching), 25% of chlorophyll molecules are within 10Å of a second chlorophyll. This is consistent with 100% quenching if the remaining 75% are able to transfer excitations to a nearby quenching pair, as was seen in Beddard and Porter’s original model (10).

A quantitatively accurate comparison with measured overall quenching rates would require a more realistic picture of the relative positions and orientations of solvated chlorophyll molecules. The sensitivity of the coupling to relative orientation means that there would be a broad range of quenching rates even within the population of closely spaced (≤10Å) chlorophyll pairs. This might, in part, be mitigated by the bulkiness of the porphyrin head group, which greatly reduces the number of possible orientations that chlorophyll pairs can adopt at very short Mg–Mg separations (13). For example, the distribution of coupling values for 8Å pairs is much narrower than for 10Å pairs (*SI Appendix*, Fig. S8). However, a full analysis of the probability of forming chlorophyll pairs with a given orientation would require larger-scale simulations of chlorophyll solutions, taking into account interactions (e.g., electrostatic attraction) that might increase the chance of chlorophyll aggregating or aligning in different solvents.

A future investigation into the distribution and geometries of chlorophyll molecules in different solvents would, furthermore, play an important role in distinguishing between different possible quenching mechanisms by determining the likelihood of forming pairs with a relative separation and orientation appropriate for charge transfer or H-aggregate formation. In their 2013 paper on quenching via H-aggregate formation, Shi et al. present Monte Carlo simulations of randomly placed chlorophyll molecules in vacuum suggesting that at 0.1mol dm^−3^, approximately 80% of chlorophyll molecules will form H-aggregates where the lowest energy excited state is a dark state (oscillator strength is close to zero) (13). By contrast, even though fixing the Mg–Mg separation favors a roughly cofacial arrangement (i.e., an H-aggregate conformation), we find that the majority of exciton states are dominated by a single monomer excited state and, therefore, will not act as dark trap states (*SI Appendix*, Fig. S9). The most likely cause of this difference is that fluctuations of the porphyrin ring and the local solvent environment break the energetic symmetry between the chlorophyll monomers assumed by Shi et al.

### Environmental Control of Quenching.

A clear conclusion of this study is that the environment (solvent or protein) plays multiple roles in facilitating or inhibiting quenching. First, solvent stabilization of the charge-separated state relative to the excited state is what makes nonradiative decay via the CS state possible. In vacuum, the charge-separated state lies too high in energy to contribute significantly to the excited state dynamics (13), while in the photosynthetic reaction center, the low-energy CT state of the special pair bacteriochlorophylls facilitates the charge separation that kickstarts the photosynthetic reaction cycle (19).

The recognition that solvents of different polarity would provide different levels of stabilization to an intermediate charge-separated state is often recognized as a strong indicator for this type of quenching mechanism (16, 23). More complex interactions, such as coordination of the central Chl magnesium atom by solvent lone pairs, could also potentially alter the relative state energies. This could be explored in greater depth by including selected solvent molecules within the quantum mechanical (QM) region of the energy calculations. However, even considering only the electrostatic (point-charge) component of this interaction, as we have done here, we would expect to see a difference in charge separation rates between coordinating and noncoordinating solvents. Moreover, the energetic cost of disrupting this type of interaction is likely to influence the range of conformations that chlorophyll pairs adopt. For example, the likelihood of forming H-aggregates could be significantly reduced if reaching a cofacial arrangement meant displacing a coordinating solvent molecule. If it could be shown (e.g., using detailed molecular dynamics simulations) that the same interactions that stabilize the charge-separated state also reduce the probability of H-aggregate formation, comparing fluorescence yields in coordinating and noncoordinating solvents would provide an excellent test for conclusively discriminating between the two possible mechanisms.

Local solvent interactions introduce a degree of geometric and energetic disorder (which likely reduces the probability of H-aggregate formation). However, it is also possible for a structured environment to impose a considerable degree of order by restricting the conformations that the chlorophyll pairs can adopt. The LH2 antenna complex is a perfect example of this. As well as raising the minimum energy of the CS state, the protein environment makes it (energetically) extremely difficult for the bacteriochlorophyll pair to reach a conformation that is favorable for charge transfer.

The overall effect is a perfect balance of allowing chromophores to be held close enough together to allow efficient exciton transfer, without any risk of wasting energy by unwanted quenching via the charge-separated state. Further investigation into the chlorophyll configurations that promote charge separation and why they can’t be reached inside the LH2 protein pocket would be extremely useful for learning how to incorporate this essential feature into the design of synthetic light-harvesting devices.

## Materials and Methods

### Geometry Sampling.

Molecular dynamics simulations were performed using OpenMM (37) for 12 different systems: a pair of neutral chlorophyll molecules (Chl – Chl) or a pair of chlorophyll ions (Chl${}^{+}$ – Chl${}^{-}$) embedded in light-harvesting complex 2 (LH2) from purple photosynthetic bacteria (*Rps. acidophila*) or solvated in diethyl ether with an Mg–Mg separation of 8, 10, 12, 14, or 20Å.

Parmed (38) was used to extract the initial geometries and forcefield parameters for solvated neutral chlorophyll from the first two Cla residues in Zhang and coworkers’ forcefield for Photosystem II (39). The vector between the central Mg atoms in each Cla residue was adjusted to the desired length and then fixed as a single constraint in the forcefield. Solvent (diethyl ether) molecules were added around the Cla residues using Packmol (40) to create a cubic unit cell with a side length of 100Å and a solvent density of 0.71g ml^−1^. Solvent parameters were taken from OpenForceField version 1.3.0 (41). The system was allowed to equilibrate for 10ps (20000 0.5fs timesteps) before the first geometry was saved. After equilibration, the simulation was run for a further 500ps, recording geometries at intervals of 1ps. The dynamics were simulated at 300 K using a Langevin Integrator. The use of this integrator imposes an NVT ensemble, and the energy differences between sample geometries are Helmholtz free energies. Since the systems being described have a low compressibility, the difference between Helmholtz and Gibbs free energies will be negligible. Consequently, we follow the example of other previous studies (e.g., ref. (42)) in using ΔA directly in place of ΔG in Eq. **1**. Simulations of LH2 were run under the same conditions, using the LH2 forcefield created by Ramos et al. (34).

Atomic partial charges $\left\{q_{i}^{\prime }\right\}$ for the chlorophyll ion forcefields were generated according to

[2] $$\begin{array}{c} q_{i}^{\prime }=q_{i}+\left(q_{i}^{\;\text{ion}}-q_{i}^{\;\text{neutral}}\right), \end{array}$$

where $q_{i}$ is the partial charge on atom i in the neutral chlorophyll forcefield; $q_{i}^{\;\text{ion}}$ is the partial charge calculated at the GFN1-xTB level of theory for a charged chlorophyll residue embedded in a point charge representation of the surroundings (using the point charges from the neutral forcefield); and $q_{i}^{\;\text{neutral}}$ is the atomic charge predicted by the equivalent GFN1-xTB calculation on a neutral chlorophyll residue.

### Free-Energy Surfaces.

The free-energy surfaces for the ground (GS) and charge-separated (CS) states were constructed from the equilibrium probability distribution of vertical energy gaps ΔE relative to a second state, sampled over a molecular dynamics trajectory, as is done in reference (24, 43). For example, to create the surfaces shown in Fig. 3, we calculated the energy of the excited state $E_{\;\text{ES}}$ and the charge-separated state $E_{\;\text{CS}}$ for each of the 500 geometry snapshots taken along the molecular dynamics trajectory described above for the (Chl${}^{+}$ – Chl${}^{-}$) ion pair to obtain a distribution of $\Delta E=E_{\;\text{ES}}-E_{\;\text{CS}}$ values.

The resulting Boltzmann distributions of energy gaps are well described by a normal distribution, $P\left(\Delta E\right)=\;\text{exp}(-{\left(\left(\Delta E-\mu \right)/\sigma \right)}^{2}/2)=\exp\left(-V\left(\Delta E\right)/kT\right)$ (*SI Appendix*, Figs. S2 and S3). Taking the logarithm (and rearranging) shows the underlying free energy surfaces that give rise to this distribution have a quadratic form

[3] $$\begin{array}{c} V\left(\Delta E\right)=\frac{\mathrm{kT}}{2}{\left(\frac{\Delta E-\mu }{\sigma }\right)}^{2}. \end{array}$$

Consequently, we define an equation for the CS surface $V_{\mathrm{CS}}$, with the form of Eq. **3** and values of the mean μ and standard deviation σ obtained by fitting the sampled ΔE distribution to a normal distribution using the norm function in the scipy.stats package (44). The ES surface can be simultaneously defined by $V_{\mathrm{ES}}\left(\Delta E\right)=V_{\mathrm{CS}}\left(\Delta E\right)+\Delta E$. However, as can be seen in Figs. 1 and 3, defining the ES surface in this way leads to considerable uncertainty around the ES minimum.

In Fig. 1, the GS surface is similarly obtained by sampling energy gaps along the neutral Chl – Chl trajectory. As discussed above, the GS and ES minima occur at similar values of ΔE but are both significantly displaced relative to the CS minimum. Sampling energy gaps along the Chl – Chl trajectory therefore offers information about the relationship between the ES and CS states away from the CS minimum. The energy gap $E_{\mathrm{ES}}-E_{\mathrm{CS}}$ correlates well with $E_{\mathrm{CS}}-E_{\mathrm{GS}}$ (*SI Appendix*, Fig. S6). By fitting the (linear) relationship between these two energy gaps, the free energy surface for the excited state $V_{\mathrm{ES}}$ can be defined relative to $V_{\mathrm{CS}}\left(E_{\mathrm{CS}}-E_{\mathrm{GS}}\right)$ as a function of $E_{\mathrm{CS}}-E_{\mathrm{GS}}$. In Figs. 1*c*, 3, and 4, we have replotted $V_{\mathrm{ES}}$ as a function of $\Delta E=E_{\mathrm{ES}}-E_{\mathrm{CS}}$.

Once the CS and ES states have been defined, the quantities required to calculate Marcus theory rate constants can be evaluated as $\Delta A=V_{\mathrm{CS}}\left(\Delta E_{\mathrm{CS}\min}\right)-V_{\mathrm{ES}}\left(\Delta E_{\mathrm{ES}\min}\right)$ and $\lambda =V_{\mathrm{CS}}\left(\Delta E_{\mathrm{ES}\min}\right)-V_{\mathrm{CS}}\left(\Delta E_{\mathrm{CS}\min}\right)$, where $\Delta E_{\mathrm{CS}\min}$ and $\Delta E_{\mathrm{ES}\min}$ are the values of ΔE at the minima of $V_{\mathrm{CS}}$ and $V_{\mathrm{ES}}$, respectively.

### Energy Evaluations.

Single-point energies of each geometry snapshot were constructed from a sequence of calculations performed on individual chlorophyll monomers. This approach was used to avoid the prohibitive cost of performing large numbers (1,500 per trajectory) of energy calculations on the whole chlorophyll pair at a sufficiently high level of theory to capture the desired charge-separated state. The ground-state energy and atomic point charges of each chlorophyll molecule (or ion) were calculated using GFN1-xTB (45) with a periodic point charge embedding. The point charges and their positions were taken from the forcefields above. To bring the computational cost within an affordable level, the LH2 unit cell was reduced to the same size as for the solvated systems by discarding any molecules that did not have at least one atom within 50Å of the original unit cell center.

The energy $E_{A}^{\mathrm{qm}/\mathrm{mm}}=E_{A}^{\mathrm{xTB},\mathrm{pol}}+E_{A,s}+E_{s,s}$ returned by each embedded monomer calculation is a sum of the quantum mechanical energy of monomer A (polarized by the interaction with its surroundings), the electrostatic interaction energy $E_{A,s}$ between the atomic charges of monomer A and the surroundings (including periodic images of monomer A), and the electrostatic interaction energy $E_{s,s}$ between the point charges in the surroundings. The energy of the full system $E_{\mathrm{AB}}^{\mathrm{qm}/\mathrm{mm}}$ is subsequently calculated as the sum of $E_{A}^{\mathrm{xtb},\;\mathrm{pol}}$ for each monomer, plus an electrostatic interaction between all pairs of atomic charges in the system, including the newly calculated (xTB-level) charges for each chlorophyll monomer and the forcefield charges for the solvent. Detailed equations for the final energy are given in *SI Appendix*.

Monomer excitation energies and transition charges were calculated using a bespoke semiempirical method, designed by us to efficiently describe the $Q_{y}$ transition of chlorophyll with an accuracy comparable to PBE0/Def2-SVP TDDFT (for single bacteriochlorophyll a monomers, it produces excitation energies with an RMSE of 0.014 eV and a coefficient of correlation of 0.88 compared to PBE0/def2-SVP; transition dipoles with RMSE of 0.057 a.u. and correlation coefficient of 0.4; and exciton energies for a BChla pair with an RMSE of 0.047 eV and coefficient of correlation of 0.806). Full details and benchmarking of this method will be published separately but a brief overview of the key equations are given in *SI Appendix*. The method centers around the idea that since chlorophyll’s $Q_{y}$ transition is dominated by a single orbital excitation, its energy can be approximated by the corresponding diagonal element of the ‘A’ matrix in the standard linear response (Casida) equations (46). This matrix element is constructed using the framework that underpins Grimme and Bannworth’s sTDA-xTB (simplified Tamm–Dancoff approximation with extended tight-binding) approach (47), with several parameters refitted to better describe the desired transition.

The excited state energy of the chlorophyll pair was calculated using a Frenkel exciton Hamiltonian,

[4] $$\hat{H}=\sum_{i}E_{i}|i\rangle \;\langle i|+\sum_{i\neq j}V_{ij}|j\rangle \;\langle j|,$$

where $E_{i}$ is a shorthand denoting the energy of an (embedded) chlorophyll pair (i.e., $E_{\mathrm{AB}}^{\mathrm{qm}/\mathrm{mm}}$) with monomer i in its excited state and $V_{\mathrm{ij}}$ is the coulomb interaction between the transition charges associated with excitation on each chlorophyll. The effect of the environment on the strength of $V_{\mathrm{ij}}$ is encoded in the transition charges, which are ultimately derived from a polarized ground state electronic structure (*SI Appendix*) that includes interactions with the solvent or protein surroundings. The lowest energy eigenvalue of Eq. **4** was used to construct the excited state free-energy surfaces.

All quantum chemical calculations were carried out using Qcore (https://software.entos.ai/qcore), with an open-source implementation of compressed particle-mesh Ewald (48). Parameters for the particle-mesh Ewald calculations are given in *SI Appendix*, Table 2.

### Coupling Elements.

The coupling $H_{\mathrm{ab}}$ between the excited and charge-separated states was calculated using the Fragment Orbital Density Functional Theory (FODFT) approach (49, 50). DFT calculations were performed using the PBE functional and 3-21++G basis set, which gave good agreement with earlier benchmark studies (33) (*SI Appendix*, Fig. S10). Although the donor and acceptor are, respectively, Chl and Chl${}^{-}$, orbitals were calculated for Chl${}^{+}$ and Chl${}^{-}$ fragments, following previous observations that better coupling values are obtained by using approximate orbitals to construct a Hamiltonian element with the correct number of electrons (51). To calculate the coupling elements, chlorophyll fragments were truncated by replacing the phytyl tail with a hydrogen atom to allow calculations at the required level of theory. This truncation is not expected to alter the results, since the frontier orbitals are strongly centered around the porphyrin ring of the chlorophyll molecule. For all other calculations (i.e., QM/MM calculations of energies), the full chlorophyll molecule was kept without truncation. The accuracy of FODFT coupling values is highly sensitive to basis set truncation errors and thus decreases noticeably at large fragment separations. For this reason, we did not directly calculate $H_{\mathrm{ab}}$ values for chlorophyll separations larger than 10Å.

## Supplementary Material

Appendix 01 (PDF)

Dataset S01 (XLSX)

Dataset S02 (TXT)

Dataset S03 (TXT)

Dataset S04 (TXT)

Dataset S05 (TXT)

Dataset S06 (TXT)

Dataset S07 (TXT)

Dataset S08 (TXT)

Dataset S09 (TXT)

Dataset S10 (TXT)

Dataset S11 (TXT)

Dataset S12 (TXT)

Dataset S13 (TXT)

Dataset S14 (TXT)

Dataset S15 (TXT)

Dataset S16 (TXT)

Dataset S17 (TXT)

Dataset S18 (TXT)

Dataset S19 (TXT)

## Data Availability

All study data are included in the article and/or *SI Appendix*.

## References

[1] W. F. Watson, R. Livingston, Self-quenching and sensitization of fluorescence of chlorophyll solutions. J. Chem. Phys. **18**, 802–809 (1950).

[2] G. R. Seely, The energetics of electron-transfer reactions of chlorophyll and other compounds. Photochem. Photobiol. **27**, 639–654 (1978).

[3] F. Thomas , De Novo-designed α-helical barrels as receptors for small molecules. ACS Synth. Biol. **7**, 1808–1816 (2018). 29944338 10.1021/acssynbio.8b00225

[4] Q. Zou, K. Liu, M. Abbas, X. Yan, Peptide-modulated self-assembly of chromophores toward biomimetic light-harvesting nanoarchitectonics. Adv. Mater. **28**, 1031–1043 (2016). 26273821 10.1002/adma.201502454

[5] E. Meneghin , Biomimetic nanoarchitectures for light harvesting: Self-assembly of pyropheophorbide-peptide conjugates. J. Phys. Chem. Lett. **11**, 7972–7980 (2020). 32886518 10.1021/acs.jpclett.0c02138 PMC8011917

[6] A. R. Kelly, G. Porter, Model systems for photosynthesis I. Energy transfer and light harvesting mechanisms. Proc. R. Soc. London. A. Math. Phys. Sci. **315**, 149–161 (1970).

[7] A. R. Kelly, L. K. Patterson, Model systems for photosynthesis II. Concentration quenching of chlorophyll b fluorescence in solid solutions. Proc. R. Soc. London. A. Math. Phys. Sci. **324**, 117–126 (1971).

[8] G. Beddard, S. Carlin, G. Porter, Concentration quenching of chlorophyll fluorescence in bilayer lipid vesicles and liposomes. Chem. Phys. Lett. **43**, 27–32 (1976).

[9] M. J. Yuen, L. L. Shipman, J. J. Katz, J. C. Hindman, Concentration quenching of fluorescence from chlorophyll-a, pheophytin-a, pyropheophytin-a and their covalently-linked pairs. Photochem. Photobiol. **32**, 281–296 (1980).

[10] G. S. Beddard, G. Porter, Concentration quenching in chlorophyll. Nature **260**, 366–367 (1976).

[11] V. P. Gutschick, Concentration quenching in chlorophyll-a and relation to functional charge transfer in vivo. J. Bioenerg. Biomembr. **10**, 153–170 (1978). 555463 10.1007/BF00743105

[12] T. P. J. Krüger, R. van Grondelle, The role of energy losses in photosynthetic light harvesting. J. Phys. B: Atomic, Mol. Opt. Phys. **50**, 132001 (2017).

[13] W. J. Shi, J. Barber, Y. Zhao, Role of formation of statistical aggregates in chlorophyll fluorescence concentration quenching. J. Phys. Chem. B **117**, 3976–3982 (2013). 23514224 10.1021/jp311821t

[14] R. S. Knox, Spectral effects of exciton splitting in “statistical pairs’’. J. Phys. Chem. **98**, 7270–7273 (1994).

[15] K. K. Mentel, R. M. D. Nunes, C. Serpa, L. G. Arnaut, Dynamics of radical ion pairs following photoinduced electron transfer in solvents with low and intermediate polarities. J. Phys. Chem. B **119**, 7571–7578 (2015). 25588979 10.1021/jp511425y

[16] C. McCleese , Excitonic interactions in bacteriochlorin homo-dyads enable charge transfer: A new approach to the artificial photosynthetic special pair. J. Phys. Chem. B **122**, 4131–4140 (2018). 29526105 10.1021/acs.jpcb.8b02123 PMC6422163

[17] A. Dreuw, G. R. Fleming, M. Head-Gordon, Chlorophyll fluorescence quenching by xanthophylls. Phys. Chem. Chem. Phys. **5**, 3247 (2003).

[18] A. Warshel, W. W. Parson, Spectroscopic properties of photosynthetic reaction centers 1 theory. J. Am. Chem. Soc. **109**, 6143–6152 (1987).

[19] W. W. Parson, A. Warshel, Spectroscopic properties of photosynthetic reaction centers. 2. Application of the theory to rhodopseudomonas viridis. J. Am. Chem. Soc. **109**, 6152–6163 (1987).

[20] R. E. Overfield, A. Scherz, K. J. Kaufmann, M. R. Wasielewski, Photophysics of bis(chlorophyll)cyclophanes: Models of photosynthetic reaction centers. J. Am. Chem. Soc. **105**, 4256–4260 (1983).

[21] M. R. Wasielewski, M. P. Niemczyk, Photoinduced electron transfer in meso-triphenyltriptycenylporphyrin-quinones. Restricting donor-acceptor distances and orientations. J. Am. Chem. Soc. **106**, 5043–5045 (1984).

[22] H. S. Kang , Effects of strong electronic coupling in chlorin and bacteriochlorin dyads. J. Phys. Chem. A **120**, 379–395 (2016). 26765839 10.1021/acs.jpca.5b10686

[23] M. R. Wasielewski , Chlorophyll-porphyrin heterodimers with orthogonal systems: Solvent polarity dependent photophysics. J. Am. Chem. Soc. **112**, 6482–6488 (1990).

[24] A. Warshel, W. W. Parson, Dynamics of biochemical and biophysical reactions: Insight from computer simulations. Q. Rev. Biophys. **34**, 563–679 (2001). 11852595 10.1017/s0033583501003730

[25] R. A. Kuharski , Molecular model for aqueous ferrous-ferric electron transfer. J. Chem. Phys. **89**, 3248–3257 (1988).

[26] L. L. Shipman, T. M. Cotton, J. R. Norris, J. J. Katz, An analysis of the visible absorption spectrum of chlorophyll a monomer, dimer, and oligomers in solution. J. Am. Chem. Soc. **98**, 8222–8230 (1976). 993521 10.1021/ja00441a056

[27] L. Hedayatifar , Optical absorption and electronic spectra of chlorophylls a and b. RSC Adv. **6**, 109778–109785 (2016).

[28] S. S. Brody, Fluorescence lifetime, yield, energy transfer and spectrum in photosynthesis, 1950–1960. Photosynth. Res. **73**, 127–132 (2002). 16245113 10.1023/A:1020405921105

[29] M. Cho, R. J. Silbey, Nonequilibrium photoinduced electron transfer. J. Chem. Phys. **103**, 595–606 (1995).

[30] L. Q. Dong, K. Niu, S. L. Cong, Theoretical analysis of internal conversion pathways and vibrational relaxation process of chlorophyll-a in ethyl ether solvent. Chem. Phys. Lett. **440**, 150–154 (2007).

[31] D. M. Silver, K. Ruedenberg, Overlap integrals over slater-type atomic orbitals. J. Chem. Phys. **49**, 4301–4305 (1968).

[32] N. C. Handy, M. T. Marron, H. J. Silverstone, Long-range behavior of Hartree-Fock orbitals. Phys. Rev. **180**, 45–48 (1969).

[33] A. Kubas , Electronic couplings for molecular charge transfer: Benchmarking CDFT, FODFT and FODFTB against high-level ab initio calculations. II. Phys. Chem. Chem. Phys. **17**, 14342–14354 (2015). 25573447 10.1039/c4cp04749d

[34] F. Cardoso Ramos, M. Nottoli, L. Cupellini, B. Mennucci, The molecular mechanisms of light adaption in light-harvesting complexes of purple bacteria revealed by a multiscale modeling. Chem. Sci. **10**, 9650–9662 (2019). 32055335 10.1039/c9sc02886b PMC6988754

[35] X. Li, R. M. Parrish, F. Liu, S. I. Kokkila Schumacher, T. J. Martínez, An ab initio exciton model including charge-transfer excited states. J. Chem. Theory Comput. **13**, 3493–3504 (2017). 28617595 10.1021/acs.jctc.7b00171

[36] T. P. Fay, D. T. Limmer, Coupled charge and energy transfer dynamics in light harvesting complexes from a hybrid hierarchical equations of motion approach. J. Chem. Phys. **157**, 174104 (2022). 36347697 10.1063/5.0117659

[37] P. Eastman , OpenMM 7: Rapid development of high performance algorithms for molecular dynamics. PLOS Comput. Biol. **13**, e1005659 (2017). 28746339 10.1371/journal.pcbi.1005659 PMC5549999

[38] M. R. Shirts , Lessons learned from comparing molecular dynamics engines on the SAMPL5 dataset. J. Comput.-Aided Mol. Des. **31**, 147–161 (2017). 27787702 10.1007/s10822-016-9977-1 PMC5581938

[39] L. Zhang, D. A. Silva, Y. Yan, X. Huang, Force field development for cofactors in the photosystem II. J. Comput. Chem. **33**, 1969–1980 (2012). 22685077 10.1002/jcc.23016

[40] L. Martínez, R. Andrade, E. G. Birgin, J. M. Martínez, PACKMOL: A package for building initial configurations for molecular dynamics simulations. J. Comput. Chem. **30**, 2157–2164 (2009). 19229944 10.1002/jcc.21224

[41] J. Wagner, M. Thompson, D. Dotson, Hyejang, J. Rodríguez-Guerra, openforcefield/openforcefields: Version 1.3.0 “Parsley” Update (2020).

[42] J. Blumberger, Recent advances in the theory and molecular simulation of biological electron transfer reactions. Chem. Rev. **115**, 11191–11238 (2015). 26485093 10.1021/acs.chemrev.5b00298

[43] T. A. Barnes, J. W. Kaminski, O. Borodin, T. F. Miller, Ab initio characterization of the electrochemical stability and solvation properties of condensed-phase ethylene carbonate and dimethyl carbonate mixtures. J. Phys. Chem. C **119**, 3865–3880 (2015).

[44] P. Virtanen , SciPy 1.0: Fundamental algorithms for scientific computing in Python. Nat. Methods **17**, 261–272 (2020). 32015543 10.1038/s41592-019-0686-2 PMC7056644

[45] S. Grimme, C. Bannwarth, P. Shushkov, A robust and accurate tight-binding quantum chemical method for structures, vibrational frequencies, and noncovalent interactions of large molecular systems parametrized for all spd-Block elements ( Z = 1–86). J. Chem. Theory Comput. **13**, 1989–2009 (2017). 28418654 10.1021/acs.jctc.7b00118

[46] M. E. Casida, Time-Dependent Density Functional Response Theory for Molecules in Recent Advances in Density Functional Methods, Part 1 (1995), pp. 155–192.

[47] S. Grimme, C. Bannwarth, Ultra-fast computation of electronic spectra for large systems by tight-binding based simplified Tamm-Dancoff approximation (sTDA-xTB). J. Chem. Phys. **145**, 054103 (2016). 27497535 10.1063/1.4959605

[48] A. C. Simmonett, B. R. Brooks, A compression strategy for particle mesh Ewald theory. J. Chem. Phys. **154**, 054112 (2021). 33557541 10.1063/5.0040966 PMC7986272

[49] H. Oberhofer, J. Blumberger, Revisiting electronic couplings and incoherent hopping models for electron transport in crystalline C60 at ambient temperatures. Phys. Chem. Chem. Phys. **14**, 13846 (2012). 22858858 10.1039/c2cp41348e

[50] H. Oberhofer, K. Reuter, J. Blumberger, Charge transport in molecular materials: An assessment of computational methods. Chem. Rev. **117**, 10319–10357 (2017). 28644623 10.1021/acs.chemrev.7b00086

[51] C. Schober, K. Reuter, H. Oberhofer, Critical analysis of fragment-orbital DFT schemes for the calculation of electronic coupling values. J. Chem. Phys. **144**, 054103 (2016). 26851904 10.1063/1.4940920

