# Supplementary material for: Charge transfer as a mechanism for chlorophyll fluorescence concentration quenching
Source: Proc Natl Acad Sci U S A. 2023 Jan 23;120(5):e2210811120. doi: 10.1073/pnas.2210811120 (PMC9945999; doi:10.1073/pnas.2210811120)
Supplement: Supplementary file 1 — Appendix 01 (PDF) [file pnas.2210811120.sapp.pdf]

PNAS
www.pnas.org

Supplementary Information for

Charge transfer as a mechanism for chlorophyll fluorescence concentration quenching

Susannah Bourne-Worster, Oliver Feighan, Frederick R. Manby

Susannah Bourne-Worster
E-mail: susannah.bourne-worster@bristol.ac.uk

This PDF file includes:

Supplementary text
Figs. S1 to S10
Tables S1 to S4
Legends for Dataset S1 to S19
SI References

Other supplementary materials for this manuscript include the following:

Datasets S1 to S19

Supporting Information Text

Probability of statistical pairs. The radial distribution function describes the distribution of molecules in a solution and is defined by

$$g(r) = \frac{\rho(r)}{\rho} = \frac{\text{local density}}{\text{density}}.$$

To get a back-of-the-envelope estimate of the probability of forming statistical pairs, we make the naive assumption that there is an equal probability of finding a molecule at any given point in space and therefore $g(r)$ is 1.

The number of molecules in a spherical shell of thickness dr at distance r is$N = 4\pi\rho r^2 dr$. If N is much less than 1, this is also the probability of finding a molecule in the shell (if there is half a molecule in the shell, there is a 50% chance of finding the molecule in the shell). Summing over lots of shells (ie. integrating over r) gives the probability of finding a molecule within a finite distance of the reference point (first molecule in the statistical pair):

$$\text{Prob. of molecule within distance d} = \int_0^d \rho \times g(r) \times 4\pi r^2 dr = \rho \times \frac{4/3}{\pi} d^3. \quad [1]$$

In other words, in our simplistic calculation N is simply density times the volume of the sphere that contains all points within a critical distance of the reference molecule.

To make the estimate more realistic, we consider that each chlorophyll molecule occupies a finite amount of space, creating an excluded volume that cannot be occupied by a second molecule. We define this excluded volume as $V_{\text{ex}} = \frac{4}{3}\pi r_{\text{g}}^3$, where $r_{\text{g}} = \sqrt{\sum_i^N |r_i - r_{\text{centre-of-mass}}|^2/N} = 0.96\,\text{nm}$ is the radius of gyration of chlorophyll. Taking the excluded volume into account increases the effective density of the remaining solution, since there is less space for other molecules to occupy. The new effective density is calculated as

$$\rho_{\text{eff}} = \frac{1}{\frac{1}{\rho} - V_{\text{ex}}} \quad [2]$$

and it is this that is used in equation 1 to calculate the probability of finding a second molecule within a critical distance.

Watson and Livingstone (1) measure the half-quenching concentration of chlorophyll in diethyl ether as $0.014\,\text{mol}\,\text{dm}^{-3}$. At this concentration, the probability of finding a second chlorophyll molecule within 10 Å of the first is 4%. 50% of those molecules will be found within 8 Å of each other.

Full quenching occurs at a concentration of $0.1\,\text{mol}\,\text{dm}^{-3}$, where the probability of forming a 10 Å statistical pair is 25%. At the other extreme, quenching is not observed at concentrations less that $0.001\,\text{mol}\,\text{dm}^{-3}$, where only 0.3% of molecules will form 10 Å pairs.

These rough calculations do not account for interactions that would increase the probability of finding chlorophyll molecules close together. However, they do confirm that the probability of forming statistical pairs is non-negligible and that most chromophores will be close enough to pass their energy to a quenching pair.

Dimer energies. A periodic embedded monomer calculation (QM region = chlorophyll chromophore A; MM region = point charge representation of chlorophyll chromophore B and all solvent molecules S) returns an energy comprising the following contributions:

$$
\begin{aligned}
E_{\mathrm{A}}^{\mathrm{qm/mm}} = E_{\mathrm{A}}^{\mathrm{xTB,\ pol}} + \frac{1}{2}\Bigg(& \sum_{i\in\mathrm{A}^{(0)}}\sum_{j\in\mathrm{B}^{(0)}} \frac{q_i^{\mathrm{qm}} q_j^{\mathrm{mm}}}{r_i - r_j} + \sum_{i\in\mathrm{A}^{(0)}}\sum_{j\in\mathrm{S}^{(0)}} \frac{q_i^{\mathrm{qm}} q_j^{\mathrm{mm}}}{r_i - r_j} \\
& + \sum_{i\in\mathrm{B}^{(0)}}\sum_{j\in\mathrm{A}^{(0)}} \frac{q_i^{\mathrm{mm}} q_j^{\mathrm{qm}}}{r_i - r_j} + \sum_{i\in\mathrm{B}^{(0)}}\sum_{j\in\mathrm{B}^{(0)}} \frac{q_i^{\mathrm{mm}} q_j^{\mathrm{mm}}}{r_i - r_j} + \sum_{i\in\mathrm{B}^{(0)}}\sum_{j\in\mathrm{S}^{(0)}} \frac{q_i^{\mathrm{mm}} q_j^{\mathrm{mm}}}{r_i - r_j} \\
& + \sum_{i\in\mathrm{S}^{(0)}}\sum_{j\in\mathrm{A}^{(0)}} \frac{q_i^{\mathrm{mm}} q_j^{\mathrm{qm}}}{r_i - r_j} + \sum_{i\in\mathrm{S}^{(0)}}\sum_{j\in\mathrm{B}^{(0)}} \frac{q_i^{\mathrm{mm}} q_j^{\mathrm{mm}}}{r_i - r_j} + \sum_{i\in\mathrm{S}^{(0)}}\sum_{j\in\mathrm{S}^{(0)}} \frac{q_i^{\mathrm{mm}} q_j^{\mathrm{mm}}}{r_i - r_j} \\
& + \sum_{R\neq 0}\Bigg[\sum_{i\in\mathrm{A}^{(0)}}\sum_{j\in\mathrm{A}^{(R)}} \frac{q_i^{\mathrm{qm}} q_j^{\mathrm{qm}}}{r_i - r_j} + \sum_{i\in\mathrm{A}^{(0)}}\sum_{j\in\mathrm{B}^{(R)}} \frac{q_i^{\mathrm{qm}} q_j^{\mathrm{mm}}}{r_i - r_j} + \sum_{i\in\mathrm{A}^{(0)}}\sum_{j\in\mathrm{S}^{(R)}} \frac{q_i^{\mathrm{qm}} q_j^{\mathrm{mm}}}{r_i - r_j} \\
& + \sum_{i\in\mathrm{B}^{(0)}}\sum_{j\in\mathrm{A}^{(R)}} \frac{q_i^{\mathrm{mm}} q_j^{\mathrm{qm}}}{r_i - r_j} + \sum_{i\in\mathrm{B}^{(0)}}\sum_{j\in\mathrm{B}^{(R)}} \frac{q_i^{\mathrm{mm}} q_j^{\mathrm{mm}}}{r_i - r_j} + \sum_{i\in\mathrm{B}^{(0)}}\sum_{j\in\mathrm{S}^{(R)}} \frac{q_i^{\mathrm{mm}} q_j^{\mathrm{mm}}}{r_i - r_j} \\
& + \sum_{i\in\mathrm{S}^{(0)}}\sum_{j\in\mathrm{A}^{(R)}} \frac{q_i^{\mathrm{mm}} q_j^{\mathrm{qm}}}{r_i - r_j} + \sum_{i\in\mathrm{S}^{(0)}}\sum_{j\in\mathrm{B}^{(R)}} \frac{q_i^{\mathrm{mm}} q_j^{\mathrm{mm}}}{r_i - r_j} + \sum_{i\in\mathrm{S}^{(0)}}\sum_{j\in\mathrm{S}^{(R)}} \frac{q_i^{\mathrm{mm}} q_j^{\mathrm{mm}}}{r_i - r_j} \Bigg]\Bigg).
\end{aligned}
\tag{3}
$$

In equation 3, $E_{\mathrm{A}}^{\mathrm{xTB,\ pol}} = \langle\psi^{\mathrm{emb}}|H|\psi^{\mathrm{emb}}\rangle$ is the pure quantum mechanical energy of the chlorophyll chromophore A (calculated using GFN1-xTB), which has been polarised by the interaction with the point charge surroundings. The remaining terms are Coulomb interactions between the shell-resolved charges q_i^{qm} on chlorophyll A (generated by the xTB calculation) and the point charges q_j^{mm} of the surroundings. The factor of 1/2 accounts for the fact that each interaction is double-counted in the sum. The periodic nature of the calculation is highlighted by separating each interaction into a contribution from the interaction between a pair of charges both in the unit cell ($R = 0$) and a contribution from the interaction of a charge in the unit cell interacting with the periodic images ($R \neq 0$) of the second charge.

An embedded monomer calculation, as described above, was performed for each chlorophyll molecule individually. From each calculation, the polarised QM energy $E_{\mathrm{A/B}}^{\mathrm{xTB,\ pol}}$ and the set of shell-resolved charges $q_{\mathrm{A/B}}^{\mathrm{qm}}$ were saved and used to calculate the energy of the full chlorophyll pair according to

$$
\begin{aligned}
E_{\mathrm{AB}}^{\mathrm{qm/mm}} = {} & E_{\mathrm{A}}^{\mathrm{xTB,\ pol}} + E_{\mathrm{B}}^{\mathrm{xTB,\ pol}} \\
& + \frac{1}{2}\Bigg(\sum_{i\in\mathrm{A}^{(0)}}\sum_{j\in\mathrm{B}^{(0)}} \frac{q_i^{\mathrm{qm}} q_j^{\mathrm{qm}}}{r_i - r_j} + \sum_{i\in\mathrm{A}^{(0)}}\sum_{j\in\mathrm{S}^{(0)}} \frac{q_i^{\mathrm{qm}} q_j^{\mathrm{mm}}}{r_i - r_j} \\
& + \sum_{i\in\mathrm{B}^{(0)}}\sum_{j\in\mathrm{A}^{(0)}} \frac{q_i^{\mathrm{qm}} q_j^{\mathrm{qm}}}{r_i - r_j} + \sum_{i\in\mathrm{B}^{(0)}}\sum_{j\in\mathrm{S}^{(0)}} \frac{q_i^{\mathrm{qm}} q_j^{\mathrm{mm}}}{r_i - r_j} \\
& + \sum_{i\in\mathrm{S}^{(0)}}\sum_{j\in\mathrm{A}^{(0)}} \frac{q_i^{\mathrm{mm}} q_j^{\mathrm{qm}}}{r_i - r_j} + \sum_{i\in\mathrm{S}^{(0)}}\sum_{j\in\mathrm{B}^{(0)}} \frac{q_i^{\mathrm{mm}} q_j^{\mathrm{qm}}}{r_i - r_j} + \sum_{i\in\mathrm{S}^{(0)}}\sum_{j\in\mathrm{S}^{(0)}} \frac{q_i^{\mathrm{mm}} q_j^{\mathrm{mm}}}{r_i - r_j} \\
& + \sum_{R\neq 0}\Bigg[\sum_{i\in\mathrm{A}^{(0)}}\sum_{j\in\mathrm{A}^{(R)}} \frac{q_i^{\mathrm{qm}} q_j^{\mathrm{qm}}}{r_i - r_j} + \sum_{i\in\mathrm{A}^{(0)}}\sum_{j\in\mathrm{B}^{(R)}} \frac{q_i^{\mathrm{qm}} q_j^{\mathrm{qm}}}{r_i - r_j} + \sum_{i\in\mathrm{A}^{(0)}}\sum_{j\in\mathrm{S}^{(R)}} \frac{q_i^{\mathrm{qm}} q_j^{\mathrm{mm}}}{r_i - r_j} \\
& + \sum_{i\in\mathrm{B}^{(0)}}\sum_{j\in\mathrm{A}^{(R)}} \frac{q_i^{\mathrm{qm}} q_j^{\mathrm{qm}}}{r_i - r_j} + \sum_{i\in\mathrm{B}^{(0)}}\sum_{j\in\mathrm{B}^{(R)}} \frac{q_i^{\mathrm{qm}} q_j^{\mathrm{qm}}}{r_i - r_j} + \sum_{i\in\mathrm{B}^{(0)}}\sum_{j\in\mathrm{S}^{(R)}} \frac{q_i^{\mathrm{qm}} q_j^{\mathrm{mm}}}{r_i - r_j} \\
& + \sum_{i\in\mathrm{S}^{(0)}}\sum_{j\in\mathrm{A}^{(R)}} \frac{q_i^{\mathrm{mm}} q_j^{\mathrm{qm}}}{r_i - r_j} + \sum_{i\in\mathrm{S}^{(0)}}\sum_{j\in\mathrm{B}^{(R)}} \frac{q_i^{\mathrm{mm}} q_j^{\mathrm{qm}}}{r_i - r_j} + \sum_{i\in\mathrm{S}^{(0)}}\sum_{j\in\mathrm{S}^{(R)}} \frac{q_i^{\mathrm{mm}} q_j^{\mathrm{mm}}}{r_i - r_j} \Bigg]\Bigg).
\end{aligned}
\tag{4}
$$

A number of different but closely related energies are defined in equations 3 and 4 and equation (4) of the main text. For ease of reference, these quantities are summarised and defined in table S1.

Table S1. Definition and comparisons of energetic quantities.

Symbol	Definition
$E_{\text{A}}^{\text{qm/mm}}$	The total energy returned by an embedded (QM/MM) calculation on the full solvated chlorophyll system. The QM region consists of chromophore A (a single chlorophyll molecule) and the MM region incorporates all other chlorophyll molecules and surrounding solvent/protein. Defined in equation 3.
$E_{\text{A}}^{\text{xTB, pol}}$	This is the purely quantum mechanical contribution to the energy $E_{\text{A}}^{\text{qm/mm}}$. It is the expectation value of the Hamiltonian with respect to the wavefunction (ground or excited state, as required) returned by the qm/mm calculation. In this respect, it contains the effects of polarization by the surroundings (since the wavefunction has been optimized taking into account interactions with the molecules of the MM region) but it does not include the Coulombic interaction energy between QM and MM atoms or the energy of the MM region.
$E_{\text{AB}}^{\text{qm/mm}}$	The total embedded energy of a solvated chlorophyll pair. It is obtained by adding together the relative contributions from two embedded monomer calculations ($E_{\text{A}}^{\text{xTB, pol}}$, $E_{\text{B}}^{\text{xTB, pol}}$ and Coulombic point charge interactions between the quantum mechanical charges on monomers A and B and the forcefield point charges of the solvent). Defined in equation 4.
E_i	Used in equation 4 of the main text. It is the energy of a solvated chlorophyll pair, where chlorophyll i is in an excited state. Equivalent to $E_{\text{AB}}^{\text{qm/mm}}$ where the contribution $E_{\text{A}=i}^{\text{xTB, pol}}$ is an excited state energy and $E_{\text{B}\neq i}^{\text{xTB, pol}}$ is a ground state energy.

Chl-xTB method. The transition energies for each chlorophyll geometry were calculated using a bespoke semiempirical method designed to capture the Q_y transition of chlorophyll accurately and efficiently. Full details of this method will be published elsewhere but a brief outline is given here.

In linear-response TDDFT, transitions are found as the solutions to the Casida equations(2, 3)

$$\begin{pmatrix} \mathbf{A} & \mathbf{B} \\ \mathbf{B}^* & \mathbf{A}^* \end{pmatrix} \begin{pmatrix} \mathbf{X} \\ \mathbf{Y} \end{pmatrix} = \omega \begin{pmatrix} 1 & 0 \\ 0 & -1 \end{pmatrix} \begin{pmatrix} \mathbf{X} \\ \mathbf{Y} \end{pmatrix}, \quad [5]$$

where $\mathbf{A}$ and $\mathbf{B}$ are Hessian matrices describing the second derivative of the electronic energy with respect to single excitations between (Kohn-Sham) basis orbitals. The transition vectors $\mathbf{X}$ and $\mathbf{Y}$ describe each transition as a linear combination of these single excitations with a corresponding transition energy ω. Chl-xTB makes the assumption that the transitions are not strongly coupled and can therefore be approximated by the diagonal elements of $\mathbf{A}$, which for a global hybrid density functional are given by

$$\omega_{i\to a} \approx A_{ia,ia} = (\varepsilon_a - \varepsilon_i) + 2(ia|ia) - a_x(ii|aa) + (1 - a_x)(ia|f_{\mathrm{XC}}|ia). \quad [6]$$

Equation 6 tells us that the energy is the difference between individual orbital energies ε_a and ε_i, plus the response of the ground-state Coulomb integrals and the exchange-correlation functional, which contains an amount a_x of non-local Fock exchange and $1 - a_x$ of (semi-)local density functional exchange. The set of occupied orbitals are indexed i and unoccupied orbitals indexed a.

Neglecting the off-diagonal elements of $\mathbf{A}$ is similar to the single-pole approximation of Gross et al. (4, 5), as well as single transition methods such as Δ-SCF and simple eigenvalue difference approximations to excited state energies. We found these approaches overestimate the transition dipole moments of chlorophylls (by a factor of about 1.6) because the diagonal elements of $\mathbf{A}$ alone are unable to describe the mixing of Q_x and Q_y transitions. Since this is a predictable and systematic error, it is overcome in the present method by introducing an optimised scaling factor D_{scale} to the transition density (from which transition charges are derived using Mulliken population analysis).

Efficient evaluation of the diagonal elements (transition energies) is achieved using the same approximations that underpin the sTDA-xTB (simplified Tamm-Dancoff approximation with extended tight-binding) method (6): the (semi-)local exchange contribution $(1 - a_x)(ia|f_{\mathrm{XC}}|ia)$ is neglected and the remaining two-electron integrals are approximated as parameterised point-charge interactions between transition charges q_{ia}. This gives

$$\omega_{i\to a} = (\varepsilon_a - \varepsilon_i) + \sum_{\mathrm{A,B}}^{N} \left(2q_{ia}^{\mathrm{A}}\Gamma_{\mathrm{AB}}^{\mathrm{K}}q_{ia}^{\mathrm{B}} - q_{ii}^{\mathrm{A}}\Gamma_{\mathrm{AB}}^{\mathrm{J}}q_{aa}^{\mathrm{B}}\right), \quad [7]$$

where

$$\Gamma_{AB}^{J} = \left(\frac{1}{(R_{AB})^{y_J} + (a_x\eta)^{-y_J}}\right)^{\frac{1}{y_J}} \quad [8]$$

is the Mataga-Nishimoto-Ohno-Klopman Coulomb integral, and

$$\Gamma_{AB}^{K} = \left(\frac{1}{(R_{AB})^{y_K} + \eta^{-y_K}}\right)^{\frac{1}{y_K}} \quad [9]$$

is the corresponding exchange integral. The indices A and B label atoms. The optimised values of the parameters y_{K}, t_{J} and a_x are given in table S2.

The success of this semiempirical linear response method relies on having a good underlying electronic structure theory. Chl-xTB uses GFN1-xTB to generate the basis orbitals $\{i\}$ and $\{a\}$. To improve the accuracy of the transition property calculations, a small number of the GFN1-xTB parameters were reoptimised. These included the 'Hückel' constants for the s and p orbitals (k_s and k_p) and the scaling parameters for Mg and N atoms (K_n) and their interactions (K_{n-m}) (see equation 10 in reference (7)). The reoptimised values are given in table S2.

Optimisation of the semiempirical parameters was done using Sequential Least-Squares Quadratic Programming (SLSQP) with a training set of 100 random bacteriochlorophyll a geometries (sampled from a molecular dynamics trajectory for LH2), for which the transition properties had been calculated using TDDFT at the PBE0/def2-SVP level of theory (which has previously been used to simulate the LH2 absorption spectrum (8)).

Chl-xTB reproduces TDDFT transition properties extremely well for the Q_y transition in chlorophyll, with an RMSE of only 0.014 eV and a coefficient of correlation of 0.88 against PBE0/def2-SVP transition energies. Transition dipole magnitudes have an RMSE of 0.057 a.u., although a lower correlation at 0.4.

Asymmetry in the energies of the two monomers forming a Chl pair means that coupling between the chromophores only weakly influences the exciton energies at the chromophore separations that we investigate. Consequently, the larger uncertainty in transition dipole magnitudes does not translate into a similarly large uncertainty in exciton energies. Within the Frenkel exciton model (equation 4 in the main text), chl-xTB produces exciton energies with an RMSE of 0.047 eV and coefficient of correlation of 0.806 compared to PBE0/def2-SVP.

Excluded data points. Six data points (of 500) are excluded from the dataset for the 12 Å pair and one point from the dataset for the 20 Å pair. These points lie at a considerable distance from the rest of the dataset (see figure S1) and likely represent cases where the calculation has failed to converge properly. We exclude them on the basis that they disproportionately skew the fitting and bootstrapping procedures used to construct free energy surfaces and their uncertainty regions (see below).

Sampling error in FES construction. In this study, Marcus theory rate constants for charge transfer are constructed using parameters (λ and ΔA) calculated from free energy surfaces for the donor and acceptor state. These free energy surfaces are themselves constructed using the mean and standard deviation of energy gaps sampled from a molecular dynamics trajectory (see equation 3 in the main text). The standard deviation dictates the 'steepness' of the FES for the CS state (smaller standard deviation = steeper surface), while the mean dictates the position of the CS minimum along the ΔE axis. The mean and standard deviation of the sampled data are only an approximation of the underlying population statistics. By bootstrapping the sampled energies (using the `bootstrap` function in the `scipy.stats` package), we construct a 95% confidence interval (CI) on the mean and standard deviation. This gives us an indication of the variability in the shape of the free energy surfaces, arising from uncertainty in the sampling statistics. Confidence intervals calculated for each investigated chlorophyll separation are given in Table S4.

Note that the free energy surfaces are constructed using the mean and standard deviation returned by fitting function `scipy.stats.norm`, while the `scipy.stats.bootstrap` directly calculates the sample mean and standard deviation. However, since we obtain excellent fits in almost all cases (see figures S2 and S3) these are, to all intents and purposes, the same value, as shown in figure S4.

In our workflow (see main text) the shape and position of the ES surface is initially determined by the shape of the CS surface. A steeper CS surface tends to predict a smaller ΔE at the ES minimum. Combining the upper bound of the CI on the mean with the lower bound of the CI on the standard deviation gives the lowest estimates of λ and ΔA (see figure S5). The lower bound on the mean and upper bound on the standard deviation give the highest estimates of λ and ΔA. We use this combination of bounds to plot an uncertainty region around each free energy surface (red uncertainty regions on figures 1, 3, and 4 in the main text).

A better estimate of the ES surface is obtained by considering the sampled values of $E_{\mathrm{ES}} - E_{\mathrm{CS}}$ around the minimum of the GS surface (the ES minimum also lies within this region). In this part of the configuration space, $E_{\mathrm{ES}} - E_{\mathrm{CS}}$ correlates very well with $E_{\mathrm{CS}} - E_{\mathrm{GS}}$, as shown in figure S6. By fitting the linear relationship between these two energy gaps (fits given in figure S6), the ES surface can be plotted relative to the CS surface as a function of $E_{\mathrm{CS}} - E_{\mathrm{GS}}$. In an analogous way to that described above, we construct a 95% confidence interval on the gradient and intercept of the linear relationship and use the corresponding range of linear fits (noting that the more negative bound on the gradient pairs with the least negative bound on the intercept) to describe an uncertainty region around the ES surface. By carrying the bounds of this uncertainty region through the full rate constant calculation described in Materials and Methods, we determine an upper and lower bound on the values of λ, ΔA and k_{et}. These are reported in square brackets in the main text as [*lower*, *upper*].

Once all three surfaces have been defined in terms of $E_{\mathrm{CS}} - E_{\mathrm{GS}}$, the CS and ES surfaces can be replotted as a function of $E_{\mathrm{ES}} - E_{\mathrm{CS}}$ to compare the shape of the ES surfaces constructed by the two different approaches described above. In general, we find a good agreement between the two approaches, although fitting to data sampled around the ES and GS minima greatly reduces the uncertainty in the shape of the surface.

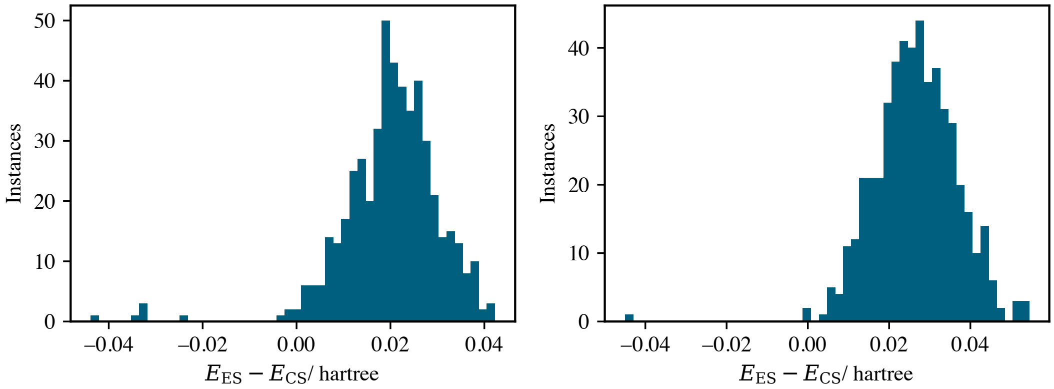

Fig. S1. Distribution of energy gaps for the solvated 12 Å (left) and 20 Å (right) separated Chl pairs. Points lying below -0.02 hartree were excluded on the basis that they disproportionately skew the fitting and bootstrapping procedures.

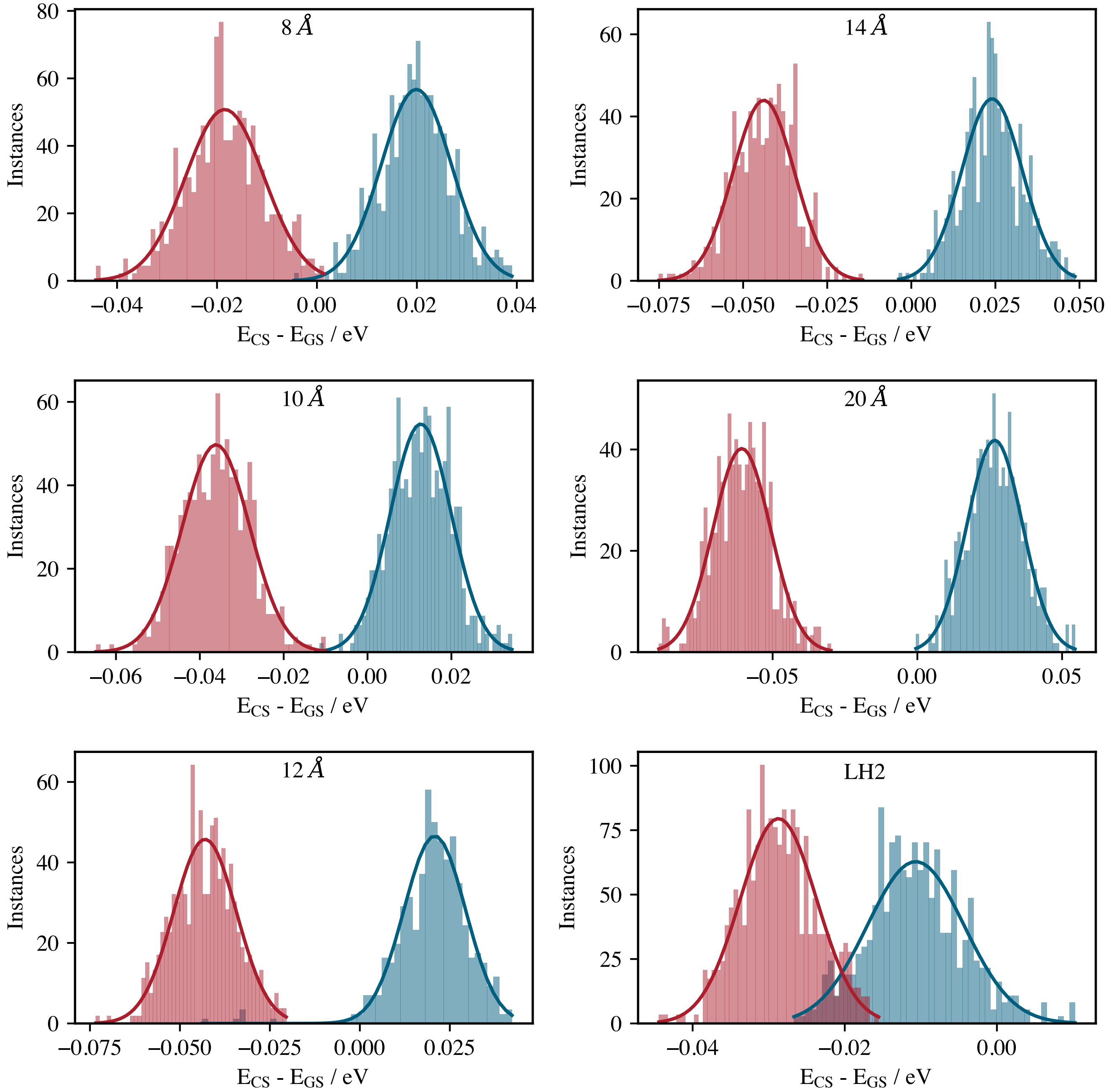

Fig. S2. Distribution and fits of ES-CS energy gaps for all Chl pairs. Red indicates energy gaps sampled by the ground state (Chl–Chl) MD trajectory and blue indicates energy gaps sampled by the charge-separated (Chl^{+}–Chl^{-}) MD trajectory. The sampled data shown in the histogram is fitted by a normal distribution. Means and standard deviations corresponding to the fitted blue lines are given in table S4.

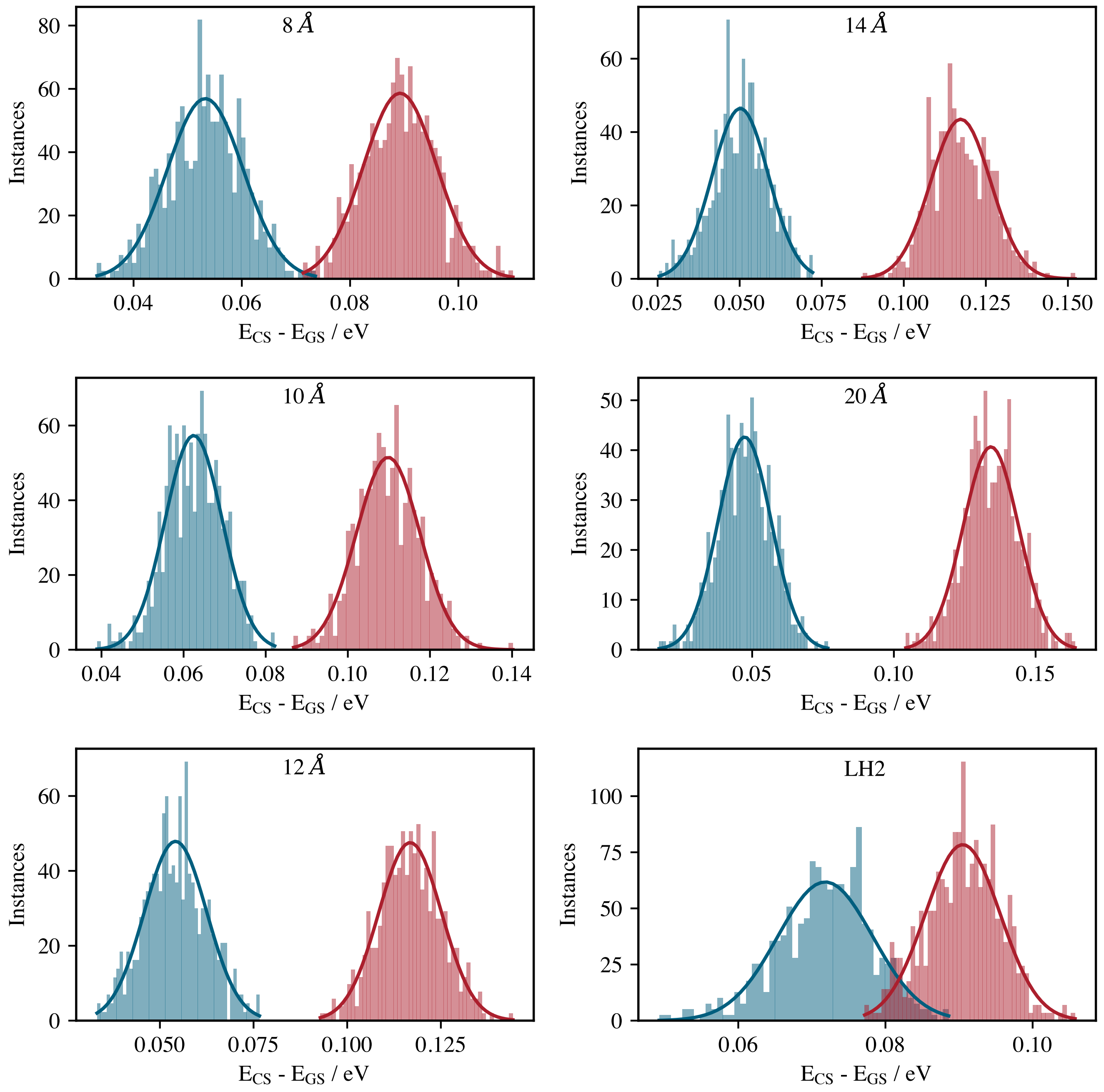

Fig. S3. Distribution and fits of GS-CS energy gaps for all Chl pairs. Red indicates energy gaps sampled by the ground state (Chl–Chl) MD trajectory and blue indicates energy gaps sampled by the charge-separated (Chl^{+}–Chl^{-}) MD trajectory. The sampled data shown in the histogram is fitted by a normal distribution.

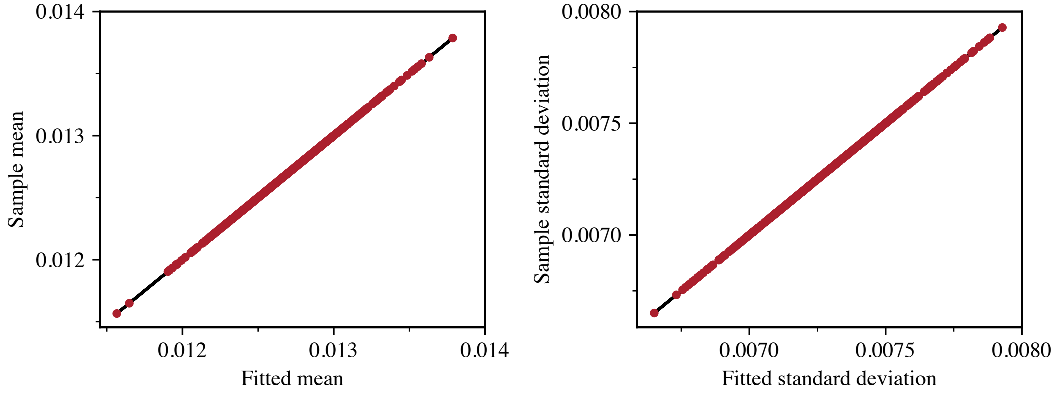

Fig. S4. Confirmation of the equality of fitted statistics and sample statistics. The LHS shows the mean μ and the RHS the standard deviation σ. Each point is generated by resampling the $(E_{\text{ES}} - E_{\text{CS}})$ dataset for the solvated $10\,\text{Å}$ Chl pair, with replacement. The mean and standard deviation are calculated directly for the resampled dataset and compared to the mean and standard deviation of a normal distribution fit to the same data.

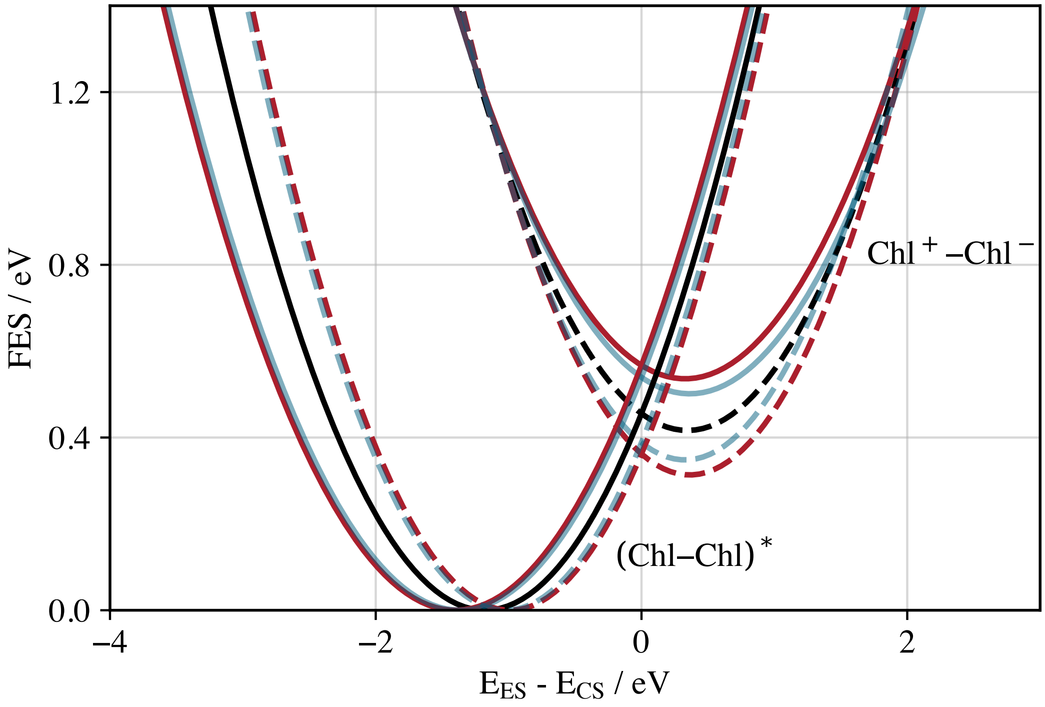

Fig. S5. Variability of the free energy surfaces for the solvated $10\,\text{Å}$ separated Chl pair. The black surfaces are constructed using the original sample mean and standard deviation. The red dashed and solid surfaces respectively use the upper (lower) bound of the confidence interval on the mean μ and lower (upper) bound on the standard deviation σ. These surfaces are used to define the shaded uncertainty regions on figure 1-3 in the main text. The dashed (solid) light blue surfaces correspond to the lower (upper) bound of the confidence interval on the mean μ and lower (upper) bound on the standard deviation σ and lie within the region defined by the red surfaces.

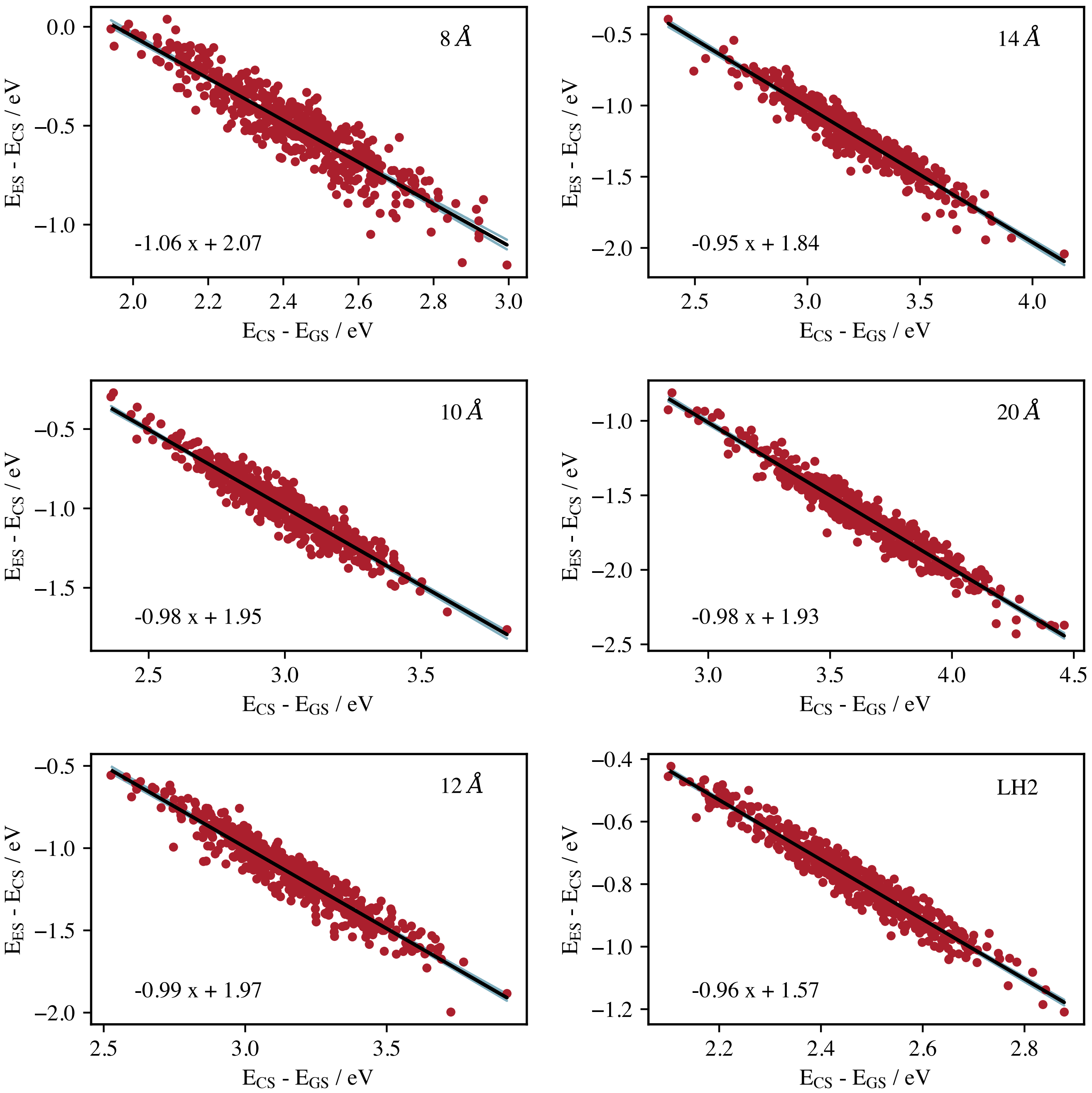

Fig. S6. Linear relationships between the ES-CS energy gap ($E_{\mathrm{ES}} - E_{CS}$) and GS-CS energy gap ($E_{\mathrm{CS}} - E_{\mathrm{GS}}$). Energy gaps were sampled at $1\,\mathrm{ps}$ intervals over a $500\,\mathrm{ps}$ molecular dynamics trajectory of an embedded pair of chlorophylls (bacteriochlorophylls in the LH2 trajectory) in their neutral ground state (Chl–Chl). Linear fits were calculated using `numpy`'s `polyfit` function. The uncertainty in the fitted relationship was determined by bootstrapping (using `scipy.stats.bootstrap`) - repeating the fitting process 9999 times by resampling the original dataset with replacement. The blue shading around the solid black line of best fit (barely visible due to the narrow uncertainty range) indicates the 95% confidence interval on the fitted line. The equation for each line of best fit is given in the lower left corner of each subplot.

Table S2. Optimised semiempirical parameters for chl-xTB

Parameter	Value in GFN1-xTB	Value in chl-xTB
k_s	1.850	1.462
k_p	2.250	2.694
K_{Mg_s}	-	1.053
K_{Mg_p}	-	0.902
K_{N_s}	-	1.281
K_{N_p}	-	1.044
$K_{\text{Mg}_s-\text{N}_s}$	-	1.468
$K_{\text{Mg}_s-\text{N}_p}$	-	1.023
$K_{\text{Mg}_p-\text{N}_s}$	-	1.067
$K_{\text{Mg}_p-\text{N}_p}$	-	1.402
y_K	2.000	2.147
y_J	4.000	4.012
a_x	0.500	0.067
D_{scale}	-	0.636

Table S3. Parameters for compressed particle-mesh Ewald calculations

Parameter	Value
Periodic box length / bohr	190.86233858720300
Ewald error tolerance	5.69167904307003E-06
α^1	0.178539963296705
Real-space cutoff	18.897300000000000000
Reciprocal-space cutoff	4.06476866978539
Spline order (9)	8
Number of FFT grid points (9)	100

[1] Reference (9) uses a parameter κ, where $\kappa^2 = \pi\alpha^2$.

Table S4. Sample statistics. Sample mean μ, standard deviation σ and their 95% confidence intervals for the distribution of $E_{\mathbf{ES}} - E_{\mathbf{CS}}$ sampled from MD trajectories for solvated/embedded Chl^{+} – Chl^{-}.

Chl-Chl separation / Å	μ	σ	Confidence interval on μ	Confidence interval on σ
8	0.0199	0.0070	[0.0205, 0.0193]	[0.0075, 0.0066]
10	0.0127	0.0073	[0.0133, 0.0120]	[0.0078, 0.0069]
12	0.0208	0.0086	[0.0216, 0.0201]	[0.0091, 0.0081]
14	0.0240	0.0090	[0.0248, 0.0232]	[0.0096, 0.0085]
20	0.0267	0.0095	[0.0276, 0.0259]	[0.0102, 0.0090]
LH2	-0.0107	0.0064	[-0.0101, -0.0112]	[0.0068, 0.0060]

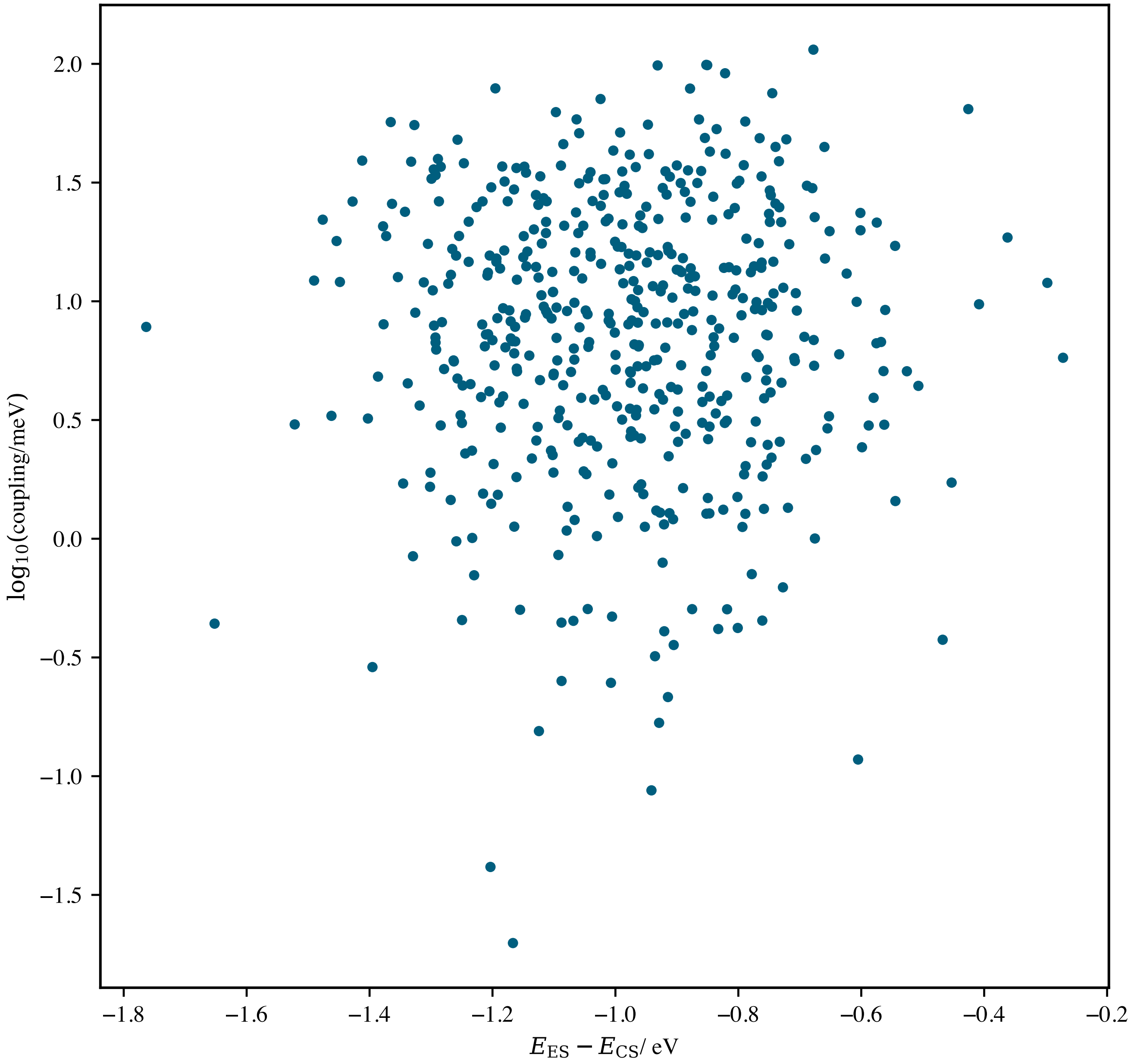

Fig. S7. Coupling $|H_{\parallel}|$ between the photoexcited and charge-separated states vs. the energy gap between these states for a $10\,\text{Å}$ separated chlorophyll pair in diethyl ether. There is no correlation between the energy gap and the value of the coupling. Details on how these quantities are calculated are found in the main text.

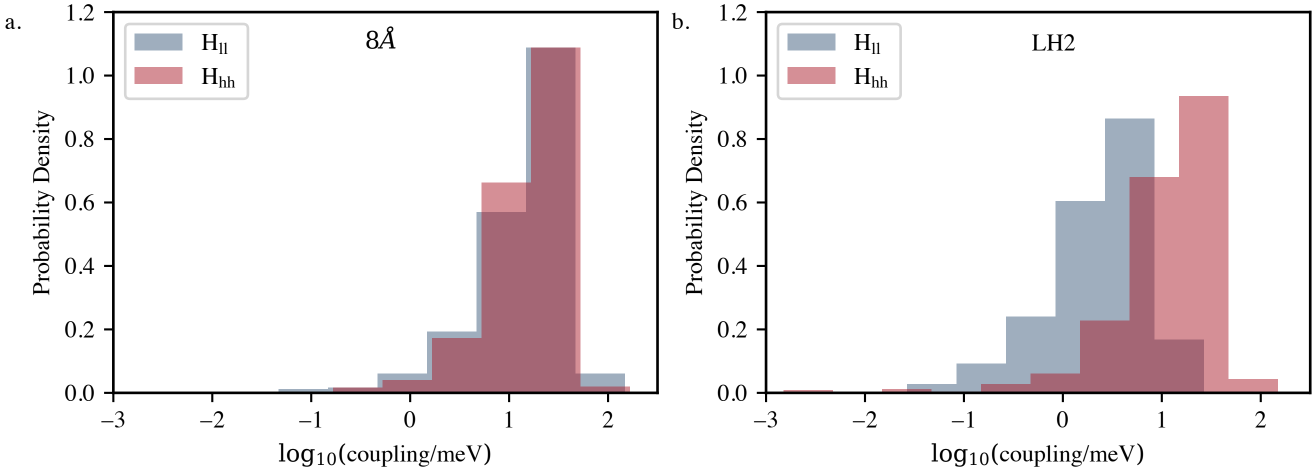

Fig. S8. Distribution of coupling values between the excited and charge-separated states of a. an $8\,\text{Å}$ and b. a $10\,\text{Å}$ separated chlorophyll pair in diethyl ether. Coupling values are calculated using FODFT at the PBE/3-21++G level on chlorophyll geometries from 500 different frames of the neutral ground state molecular dynamics trajectory, spaced $1\,\text{ps}$ apart. Two different coupling values are calculated for each geometry, corresponding to coupling between the ground state HOMO orbitals H_{hh} or ground state LUMO orbitals H_{ll} on each chlorophyll ion fragment.

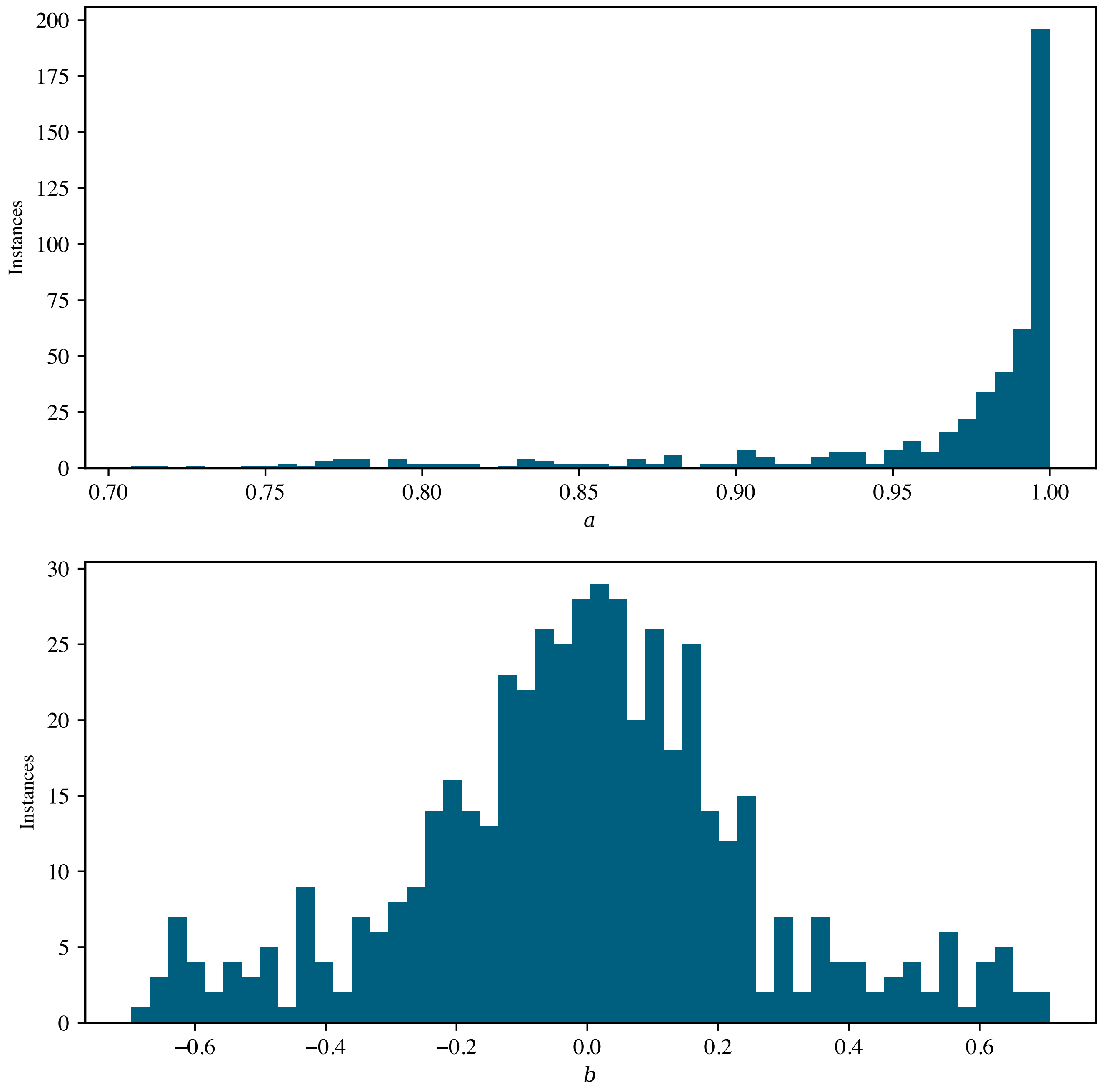

Fig. S9. The lowest energy excited states, $|\mathrm{Chl_A}\rangle$ and $|\mathrm{Chl_B}\rangle$, on individual chlorophyll monomers interact to form exciton states for the chlorophyll pair. The exciton states are calculated using an exciton hamiltonian, as described in the Methods section of the main text. The exciton states have the form $|e_1\rangle = a\,|\mathrm{Chl_A}\rangle + b\,|\mathrm{Chl_B}\rangle$. The coefficients a and b are plotted above for the exciton state with the largest component of $|\mathrm{Chl_A}\rangle$ for 500 snapshots of a neutral $10\,\text{Å}$ separated chlorophyll pair in diethyl ether. The majority of exciton states remain strongly centred on one chlorophyll monomer rather than spreading evenly over the pair.

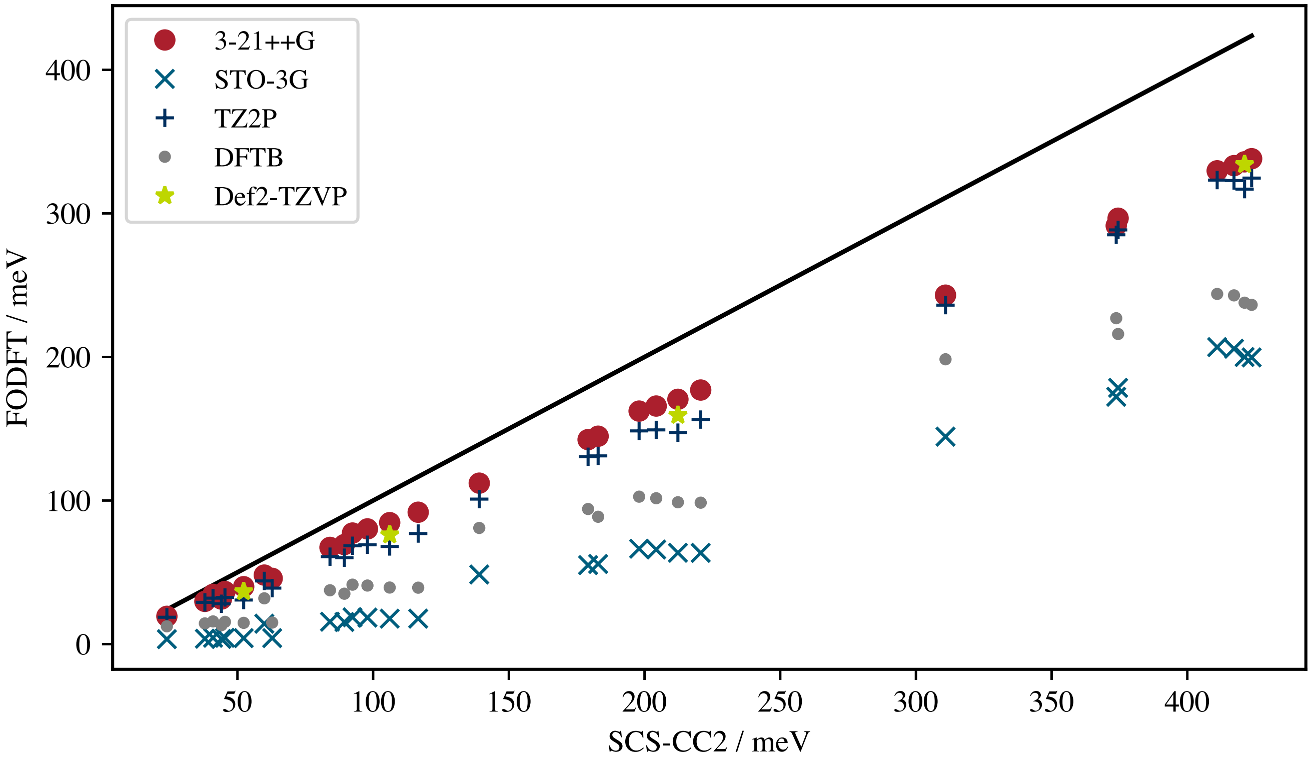

Fig. S10. Accuracy of H_{ab} values calculated using FODFT (Fragment Orbital Density Functional Theory) with the PBE functional and a range of basis sets. FODFT values are plotted against a reference value calculated using SCS-CC2 (spin-component scaled coupled cluster). Reference values and values for the TZ2P basis set and DFTB are taken from the HAB7- database (10). This test set includes dimers of anthracene, tetracene, pentacene perfluoroanthracene, perylene, perylene diimide and porphyrin with a centre-to-centre distance of $3.5\,\text{Å}$, $4.0\,\text{Å}$, $4.5\,\text{Å}$ and $5.0\,\text{Å}$.

SI Dataset S1 (EnergyData.xlsx)

Results of the energy calculations performed on the 500 individual geometry snapshots extracted from each of the 12 molecular dynamics trajectories performed. The forecefield files required to run the corresponding molecular dynamics simulations are attached in the datasets below.

SI Dataset S2 (cla_dimer_in_ether_8Asep_pdb.txt)

PDB file for a pair of Chl a molecules in diethyl ether solvent, with an 8 Å separation between Mg centres. This file contains the system geometry and can be used in conjunction with cla_dimer_in_ether_8Asep_System_xml.txt to create a forcefield in OpenMM.

SI Dataset S3 (cla_dimer_in_ether_8Asep_System_xml.txt)

XML file for a pair of Chl a molecules in diethyl ether solvent, with an 8 Å separation between Mg centres. This file contains the forcefield parameters and can be used in conjunction with cla_dimer_in_ether_8Asep_pdb.txt to create a forcefield in OpenMM.

SI Dataset S4 (cla_ion_pair_in_ether_8Asep_System_xml.txt)

XML file for a pair of Chl a ions (one cation, one anion) in diethyl ether solvent, with an 8 Å separation between Mg centres. This file contains the forcefield parameters and can be used in conjunction with cla_dimer_in_ether_8Asep_System_pdb.txt to create a forcefield in OpenMM.

SI Dataset S5 (cla_dimer_in_ether_10Asep_pdb.txt)

PDB file for a pair of Chl a molecules in diethyl ether solvent, with a 10 Å separation between Mg centres. This file contains the system geometry and can be used in conjunction with cla_dimer_in_ether_10Asep_System_xml.txt to create a forcefield in OpenMM.

SI Dataset S6 (cla_dimer_in_ether_10Asep_System_xml.txt)

XML file for a pair of Chl a molecules in diethyl ether solvent, with a 10 Å separation between Mg centres. This file contains the forcefield parameters and can be used in conjunction with cla_dimer_in_ether_10Asep_pdb.txt to create a forcefield in OpenMM.

SI Dataset S7 (cla_ion_pair_in_ether_10Asep_System_xml.txt)

XML file for a pair of Chl a ions (one cation, one anion) in diethyl ether solvent, with a 10 Å separation between Mg centres. This file contains the forcefield parameters and can be used in conjunction with cla_dimer_in_ether_10Asep_System_pdb.txt to create a forcefield in OpenMM.

SI Dataset S8 (cla_dimer_in_ether_12Asep_pdb.txt)

PDB file for a pair of Chl a molecules in diethyl ether solvent, with a 12 Å separation between Mg centres. This file contains the system geometry and can be used in conjunction with cla_dimer_in_ether_12Asep_System_xml.txt to create a forcefield in OpenMM.

SI Dataset S9 (cla_dimer_in_ether_12Asep_System_xml.txt)

XML file for a pair of Chl a molecules in diethyl ether solvent, with a 12 Å separation between Mg centres. This file contains the forcefield parameters and can be used in conjunction with cla_dimer_in_ether_12Asep_pdb.txt to create a forcefield in OpenMM.

SI Dataset S10 (cla_ion_pair_in_ether_12Asep_System_xml.txt)

XML file for a pair of Chl a ions (one cation, one anion) in diethyl ether solvent, with a 12 Å separation between Mg centres. This file contains the forcefield parameters and can be used in conjunction with cla_dimer_in_ether_12Asep_System_pdb.txt to create a forcefield in OpenMM.

SI Dataset S11 (cla_dimer_in_ether_14Asep_pdb.txt)

PDB file for a pair of Chl a molecules in diethyl ether solvent, with a 14 Å separation between Mg centres. This file contains the system geometry and can be used in conjunction with cla_dimer_in_ether_14Asep_System_xml.txt to create a forcefield in OpenMM.

SI Dataset S12 (cla_dimer_in_ether_14Asep_System_xml.txt)

XML file for a pair of Chl a molecules in diethyl ether solvent, with a 14 Å separation between Mg centres. This file contains the forcefield parameters and can be used in conjunction with cla_dimer_in_ether_14Asep_pdb.txt to create a forcefield in OpenMM.

SI Dataset S13 (cla_ion_pair_in_ether_14Asep_System_xml.txt)

XML file for a pair of Chl a ions (one cation, one anion) in diethyl ether solvent, with a 14 Å separation between Mg centres. This file contains the forcefield parameters and can be used in conjunction with cla_dimer_in_ether_14Asep_System_pdb.txt to create a forcefield in OpenMM.

SI Dataset S14 (cla_dimer_in_ether_20Asep_pdb.txt)

PDB file for a pair of Chl a molecules in diethyl ether solvent, with a 20 Å separation between Mg centres. This file contains the system geometry and can be used in conjunction with cla_dimer_in_ether_20Asep_System_xml.txt to create a forcefield in OpenMM.

SI Dataset S15 (cla_dimer_in_ether_20Asep_System_xml.txt)

XML file for a pair of Chl a molecules in diethyl ether solvent, with a 20 Å separation between Mg centres. This file contains the forcefield parameters and can be used in conjunction with cla_dimer_in_ether_20Asep_pdb.txt to create a forcefield in OpenMM.

SI Dataset S16 (cla_ion_pair_in_ether_20Asep_System_xml.txt)

XML file for a pair of Chl a ions (one cation, one anion) in diethyl ether solvent, with a 20 Å separation between Mg centres. This file contains the forcefield parameters and can be used in conjunction with cla_dimer_in_ether_20Asep_System_pdb.txt to create a forcefield in OpenMM.

SI Dataset S17 (LH2_ion_pair_pdb.txt)

PDB file for a pair of BChl a ions (one cation, one anion) in the LH2 antenna protein complex. This file contains the system geometry and can be used in conjunction with LH2_ion_pair_xml.txt to create a forcefield in OpenMM.

SI Dataset S18 (LH2_ion_pair_xml_part1.txt)

XML file for a pair of BChl a ions (one cation, one anion) in the LH2 antenna protein complex. This file contains the forcefield parameters, including atomic charges. The file is split into two parts so as not to exceed upload size - the two parts (Datasets S18 and S19) need to be recombined before use. For the corresponding forcefield parameters for the neutral LH2 system, we point the reader towards ref (11) (and thank the authors for kindly providing us with their forcefield files).

SI Dataset S19 (LH2_ion_pair_xml_part2.txt)

Second part of the XML file Dataset S18

References

1. WF Watson, R Livingston, Self-Quenching and Sensitization of Fluorescence of Chlorophyll Solutions. *The J. Chem. Phys.* **18**, 802–809 (1950).
2. ME CASIDA, Time-Dependent Density Functional Response Theory for Molecules in *Recent advances in density functional methods, part 1.* pp. 155–192 (1995).
3. F Furche, K Burke, Chapter 2 Time-Dependent Density Functional Theory in Quantum Chemistry in *Annual Reports in Computational Chemistry.* Vol. 1, pp. 19–30 (2005).
4. H Appel, EKU Gross, K Burke, Double-pole approximation in time-dependent density functional theory. *Int. J. Quantum Chem.* **106**, 2840–2847 (2006).
5. M Petersilka, UJ Gossmann, EK Gross, Excitation energies from time-dependent density-functional theory. *Phys. Rev. Lett.* **76**, 1212–1215 (1996).
6. S Grimme, C Bannwarth, Ultra-fast computation of electronic spectra for large systems by tight-binding based simplified Tamm-Dancoff approximation (sTDA-xTB). *The J. Chem. Phys.* **145**, 054103 (2016).
7. S Grimme, C Bannwarth, P Shushkov, A Robust and Accurate Tight-Binding Quantum Chemical Method for Structures, Vibrational Frequencies, and Noncovalent Interactions of Large Molecular Systems Parametrized for All spd-Block Elements (Z = 1–86). *J. Chem. Theory Comput.* **13**, 1989–2009 (2017).
8. C Stross, et al., How Static Disorder Mimics Decoherence in Anisotropy Pump–Probe Experiments on Purple-Bacteria Light Harvesting Complexes. *The J. Phys. Chem. B* **120**, 11449–11463 (2016).
9. AC Simmonett, BR Brooks, A compression strategy for particle mesh Ewald theory. *The J. Chem. Phys.* **154**, 054112 (2021).
10. A Kubas, et al., Electronic couplings for molecular charge transfer: benchmarking CDFT, FODFT and FODFTB against high-level ab initio calculations. II. *Phys. Chem. Chem. Phys.* **17**, 14342–14354 (2015).
11. FC Ramos, M Nottoli, L Cupellini, B Mennucci, The molecular mechanisms of light adaption in light-harvesting complexes of purple bacteria revealed by a multiscale modeling. *Chem. Sci.* **10**, 9650–9662 (2019).
